# Perturbations in gut microbiota composition in patients with polycystic ovary syndrome: a systematic review and meta-analysis

**DOI:** 10.1186/s12916-023-02975-8

**Published:** 2023-08-09

**Authors:** Pan Li, Ping Shuai, Sj Shen, Huimin Zheng, Ping Sun, Renfang Zhang, Shanwei Lan, Zixin Lan, Thisun Jayawardana, Yumei Yang, Jianhui Zhao, Yuping Liu, Xia Chen, Emad M. El-Omar, Zhengwei Wan

**Affiliations:** 1 Department of Obstetrics and Gynecology, The First People’s Hospital of Foshan, No.81 Lingnan Avenue North, Chancheng District, Foshan, Guangdong Province China; 2 UNSW Microbiome Research Centre, St George and Sutherland Clinical Campuses, UNSW Sydney, Clinical Sciences (WR Pitney) Building, Short St, Kogarah, NSW 2217 Australia; 3 Department of Health Management & Institute of Health Management, Sichuan Provincial People’s Hospital, University of Electronic Science and Technology of China, Chengdu, China; 4 Chinese Academy of Sciences Sichuan Translational Medicine Research Hospital, No. 32 West Second Section, First Ring Rd., Qing yang Dist, Chengdu, China; 5 The Second Clinical Medical College, Southern Medical University, Guangzhou, Guangdong Province, China; 6 Department of Big Data in Health Science School of Public Health, and Epidemiology and Biostatistics, Zhejiang University School of Medicine, Hangzhou, China

**Keywords:** Polycystic ovary syndrome, Gut microbiome, Meta-analysis, Gut dysbiosis

## Abstract

**Background:**

The results of human observational studies on the correlation between gut microbiota perturbations and polycystic ovary syndrome (PCOS) have been contradictory. This study aimed to perform the first systematic review and meta-analysis to evaluate the specificity of the gut microbiota in PCOS patients compared to healthy women.

**Methods:**

Literature through May 22, 2023, was searched on PubMed, Web of Science, Medline, Embase, Cochrane Library, and Wiley Online Library databases. Unreported data in diversity indices were filled by downloading and processing raw sequencing data. Systematic review inclusion: original studies were eligible if they applied an observational case-control design, performed gut microbiota analysis and reported diversity or abundance measures, sampled general pre-menopausal women with PCOS, and are longitudinal studies with baseline comparison between PCOS patients and healthy females. Systematic review exclusion: studies that conducted interventional or longitudinal comparisons in the absence of a control group. Two researchers made abstract, full-text, and data extraction decisions, independently. The Joanna Briggs Institute Critical Appraisal Checklist was used to assess the methodologic quality. Hedge’s g standardized mean difference (SMD), confidence intervals (CIs), and heterogeneity (*I*^2^) for alpha diversity were calculated. Qualitative syntheses of beta-diversity and microbe alterations were performed.

**Results:**

Twenty-eight studies (*n* = 1022 patients, *n* = 928 control) that investigated gut microbiota by collecting stool samples were included, with 26 and 27 studies having provided alpha-diversity and beta-diversity results respectively. A significant decrease in microbial evenness and phylogenetic diversity was observed in PCOS patients when compared with control participants (Shannon index: SMD = − 0.27; 95% CI, − 0.37 to − 0.16; phylogenetic diversity: SMD = − 0.39; 95% CI, -− 0.74 to − 0.03). We also found that reported beta-diversity was inconsistent between studies. Despite heterogeneity in bacterial relative abundance, we observed depletion of *Lachnospira* and *Prevotella* and enrichment of *Bacteroides*, *Parabacteroides*, *Lactobacillus*, *Fusobacterium*, and *Escherichia/Shigella* in PCOS. Gut dysbiosis in PCOS, which might be characterized by the reduction of short-chain fatty acid (SCFA)-producing and bile-acid-metabolizing bacteria, suggests a shift in balance to favor pro-inflammatory rather than anti-inflammatory bacteria.

**Conclusions:**

Gut dysbiosis in PCOS is associated with decreased diversity and alterations in bacteria involved in microbiota-host crosstalk.

**Trial registration:**

PROSPERO registration: CRD42021285206, May 22, 2023.

**Supplementary Information:**

The online version contains supplementary material available at 10.1186/s12916-023-02975-8.

## Background

PCOS is one of the most common female reproductive disorders and affects 5–20% of reproductive-age women worldwide [1, 2]. It is associated with a wide range of detrimental health effects, impacting reproductive, endocrine and metabolomic function [3]. However, the etiology of PCOS is controversial, and the current treatment in clinical practice relies on empirical rather than etiology-specific therapies [2]. Despite the great strides in our understanding of PCOS pathogenesis from genetic, neuroendocrine, and hormonal perspectives, we have yet to elucidate the underlying pathophysiological mechanisms. Only after understanding the initiation and development of PCOS can we then translate these findings into more effective therapeutics.

The gut microbiota is associated with a wide range of diseases, including reproductive and gynecological diseases [4–6]. There is increasing evidence to indicate that the gut microbiota interacts with the host by regulating key biological processes, such as metabolic process, hormone secretion, and immune response [7]. Indeed, dysbiosis of the gut microbiota has also been reported in microbiota-centric studies on PCOS. A recent study from Qi et al. reported that mice with fecal microbiota transplantation from patients with PCOS would develop a PCOS-like syndrome, indicating a causal role of gut microbiota in PCOS [5]. This study further demonstrated that modification of the gut microbiota, alongside bacteria-related bile acid and immune changes, may be a novel treatment for PCOS. Thus, this is an important study that shed light on both the etiology and novel therapy in PCOS.

However, many studies that contain cohorts from a range of regions and ethnicities report inconsistent and, occasionally, contradictory results. Though this variance may be explained by limited sample size and different choices of sequencing platforms, the heterogeneity among studies should be addressed to identify disease-specific bacterial biomarkers across a population, which could deepen our understanding of disease pathogenesis and would enable the development of new and effective therapies. Therefore, there is a need to compare microbial perturbations and differentiate a set of homogeneous gut microbial features from different studies. Meta-analysis is an effective method for integrating existing knowledge to uncover commonalities across different research, and interestingly, there is currently a lack of published meta-analyses that explore bacterial characteristics in PCOS patients. The present meta-analysis fills this gap in the literature by evaluating alterations in the gut microbiota of patients with PCOS across multiple studies and aims to elucidate consistent gut microbiota profiles and features with therapeutic potential.

## Methods

This study was preregistered with PROSPERO (CRD42021285206, https://www.crd.york.ac.uk/prospero/) and conducted and reported according to Preferred Reporting Items for Systematic Reviews and Meta-Analyses (PRISMA) guideline [8].

### Search strategy

A search string was developed to identify studies reporting gut microbiome and PCOS. The search strings used are available in Appendix 1 in the Additional file. In brief, we performed an extensive search of PubMed, Web of Science, Medline, Embase, Cochrane Library, and Wiley Online Library databases for articles published before May 22, 2023 (last update), that contained “Polycystic Ovary Syndrome,” “gut,” and “microbiome” in the title subheadings and main text. To ensure thorough coverage, manual searching was also performed from reviews and publication references. This study was limited to original human studies, with no language limitation.

### Eligibility criteria

Records were independently screened by two authors (ZW and HM) and any discrepancies were resolved through discussion and consultation with a third author (LP). Original studies were eligible if they (1) applied an observational case-control design, (2) performed gut microbiota analysis and reported diversity or abundance measures, (3) sampled general pre-menopausal women with PCOS, and (4) are longitudinal studies with baseline comparison between PCOS patients and healthy females. This analysis excluded studies that conducted interventional or longitudinal comparisons in the absence of a control group.

### Data extraction

Data were extracted from the selected studies by two authors (ZW and HZ) and cross-checked by two independent authors (SL and RZ) using a predesigned template. Data extraction included the following variables: study information (first author and year, study era, publication type, specimen type, diagnostic criteria of PCOS, microbiome assessment method), group information (sample number, age, body-mass index {BMI}), laboratory test indicators (total testosterone {total T}, luteinizing hormone {LH}, follicle-stimulating hormone {FSH}, ratio of LH/FSH, homeostatic model assessment for insulin resistance {HOMA-IR}), community-level measures of gut microbiota composition (alpha- and beta-diversity), and taxonomic findings at the phylum, family, and genus levels (relative abundance). Parameters of alpha-diversity, including richness (number of species) and evenness (how well each species is represented), were quantitatively extracted and, where necessary, numerical data were extracted from graphs using WebPlotDigitizer (v.4.42) [9]. Medians and inter-quartile ranges were transformed to means (M) and standard deviations (SD) using a web-based tool (http://www.math.hkbu.edu.hk/~tongt/papers/median2mean.html).

For original papers where some indices of alpha- and beta-diversity were not provided, we downloaded the raw sequencing data and calculated these indices in studies that met the following criteria: (1) the 16S rRNA gene sequencing data were available in the original publications, (2) the sequencing data were demultiplexed or barcode information for each sample was provided for demultiplex, (3) metadata were available for samples indicating whether the samples were from individuals with PCOS. The raw sequencing data of six studies were downloaded from SRA or through the links provided in the original manuscript, processed with a consistent pipeline, and the multiple indices of alpha- and beta-diversity were calculated using QIIME (version 1.9.1) [10]. For each dataset, demultiplexed sequencing reads were denoised to generate high-quality amplicon sequence variants (ASVs) using DADA2 with default parameters [11]. Taxonomy assignment of ASVs was conducted with the RDP classifier [12] against the GreenGenes database (version 13.8) [13]. The mitochondria and chloroplast ASVs were filtered. Each sample was rarefied to 8,000 sequences, and those samples with fewer than 8,000 sequences were discarded.

### Risk of bias assessment

The quality of reporting was assessed by a qualitative classification according to the Joanna Briggs Institute Critical Appraisal Checklist for Case-Control Studies, which included eight items (Additional file 1: Supplemental Table 1) [14].

### Quantitative synthesis of alpha-diversity

Meta-analysis was performed on differences in alpha-diversity between PCOS patients and controls in studies with sufficiently reported data. A random-effects meta-analysis on SMD was performed by applying the inverse-variance method. The effect size was categorized as small (SMD ≤ 0.2), moderate (SMD = 0.5), or large (SMD = 0.8). Inter-study heterogeneity was quantified using the DerSimonian-Laird estimator, reported with the *I*^2^ statistic [15]. Significant heterogeneity was defined as *I*^2^ ≥ 50% and *P* < 0.05. Pre-planned subgroup and meta regression analyses were performed to explore the determine the potential sources of heterogeneity. Pooled results and 95% CIs were further calculated with a fixed-effect model when *I*^2^ < 50%. Publication bias was evaluated with funnel plots and Egger’s regression test [16]. A trim-and-fill analysis was conducted to identify possible asymmetry and assess the robustness of the conclusions. Sensitivity analyses were conducted by (1) removing the high-risk studies and (2) removing alpha-diversity indices manually calculated by downloading original sequence data.

### Qualitative synthesis of beta-diversity and microbe alterations

Beta-diversity was qualitatively extracted to measure whether patient samples cluster significantly different compared with control participant samples. Control samples of this study were defined as healthy individuals. We took the qualitatively extracted results of beta-diversity as a dependent binary variable and performed a univariate logistic regression to explore the relationship between beta-diversity and research factors. Covariates considered for inclusion in logistic-regression models were binary variables of study era, age, BMI, and laboratory test results (total T, LH, FSH, LH/FSH, HOMA-IR) difference between the PCOS group and control group, which was extracted from the original articles.

Considering the heterogeneity of gut microbes reported in different studies, we recorded each microbe reported by the included studies in the level of phylum, family and genus, and also the species level for four shotgun metagenomic sequencing studies. A “total” row was set to summarize the overlapping microbe reported by two or more studies. A consistent finding from at least two research groups was considered potentially associated with PCOS, whereas findings only reported by one study were classified as requiring further verification.

All the analyses were performed using the R software (4.1.3). The “meta” package was utilized to finish the meta-analysis, and the “rms” package was used to finish the logistic-regression analysis. *P* < 0.05 was defined as statistically significant.

## Results

### Study selection

**Fig. 1:**
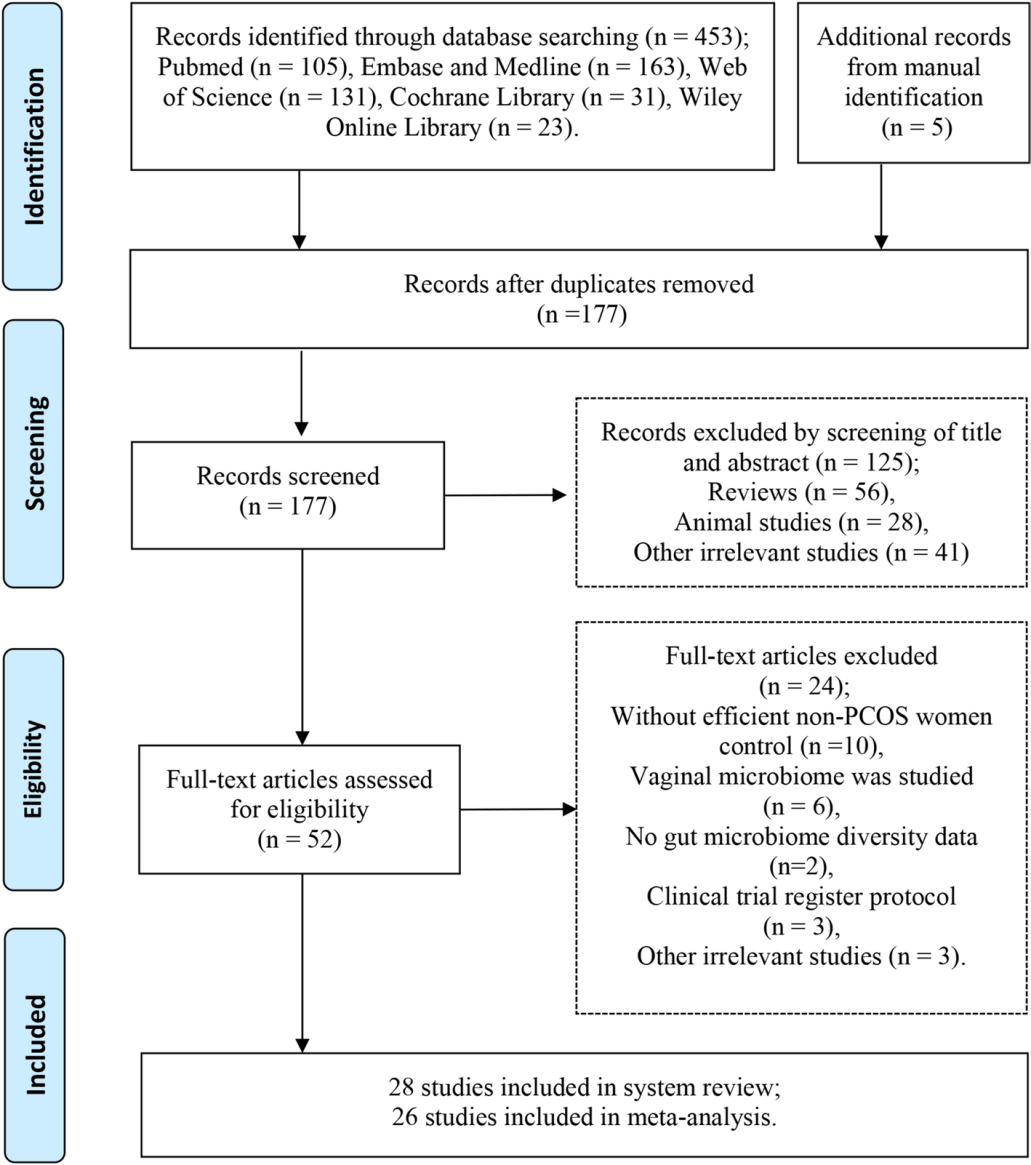
PRISMA flow diagram

According to the PRISMA searching flowcharts, we identified a total of 28 original studies, including five of which were identified by manual searching [5, 17–43] (Fig. 1). Of these included studies, 26 studies provided alpha-diversity-related indices [5, 17–21, 24–43]; 15 studies provided alpha-diversity comparisons between PCOS patients and healthy controls, which were subclassified as normal weight, over-weight, or obese according to their BMI [19, 21–23, 26–30, 32–35, 37, 41]; six studies provided original sequence data, which enabled us to re-calculate these indices [17, 18, 28, 31, 33, 37]. In total, 27 studies that compared the beta-diversity and 28 studies that compared the relative abundance of taxa were included in this meta-analysis.

### Study characteristics, risk of bias of included studies

Twenty-eight studies were included, with 26 studies having provided alpha-diversity comparison, including 118 case-control estimates (Table 1, Additional file 1: Supplemental Table 2, and Additional file 2: Supplemental Table 12). A total of ten countries/regions were covered by the included studies, with most (15, 57.69%) having been conducted in China [5, 18, 21, 27–30, 32, 33, 37, 38, 40–43]. Countries/regions were classified as either western (America, Poland, Spain, Austria, Turkey, Finland, North America, Catalan) or eastern (India, China), according to the typical diet and lifestyle. The sample size of PCOS patients across all studies ranged between seven and 102, while most studies (21, 80.77%) had small sample sizes (lower than 50) [17–19, 21–33, 35, 36, 39]. Twenty-four studies diagnosed PCOS according to Rotterdam criteria, while two studies followed NIH criteria [26, 36]. For assessment of the microbiome, most studies utilized 16S rRNA sequencing of the V3-V4 (14 studies), V1-V2 (three studies), V4 (five studies) variable regions, or full-length DNA (one study). Four studies used shotgun sequencing instead [5, 22, 23, 40]. There were substantial variations between different studies in the alpha diversity indices as measured by the Shannon index [5, 17–21, 26–31, 33–35, 37–43], Simpson index [17, 18, 27, 28, 31, 33, 37, 40], observed species [17, 18, 28, 29, 31, 33, 37, 43], Chao 1 index [17–19, 26–29, 31–33, 37, 41–43], and phylogenetic diversity (PD) whole tree [17, 18, 25, 28, 31, 33, 35, 37] (Table 1). Similarly, large variations exist in the methodology of stool processing and DNA extraction (Additional file 1: Supplemental Table 3). One of the 28 studies introduced the cohort composition of Ethnicity/Race (Caucasian, Hispanic, Black, Asian), dietary intake (fat, protein, and carbohydrate), and physical activity survey (METS) [26]. Another study calculated the Cu intake [43] (Additional file 2: Supplemental Table 12).

**Table 1:** Summary characteristics of the included studies by PCOS

Measure	No.		Total patients	Country/region of studies	Mean patient age, years	Mean patient BMI, kg/m^2^
	Studies	Estimates				
Observed species	8	10	214	China: 6; Austria: 1; Catalan: 1	15.80–29.30	20.46–29.48
Observed OTUs	11	17	302	Austria: 1; Catalan: 1; China: 6; North America: 1; Turkey: 2	15.80–29.30	21.07–33.50
Chao 1 index	14	20	400	America: 1; Austria: 1; Catalan: 1; China: 10	15.8–29.9	20.46–37.00
Shannon index	22	32	896	China: 14; America: 1; Poland: 1; Spain: 1; Austria: 1; Finland: 1; Turkey: 1; India: 1; Catalan: 1	15.80–30.00	20.46–37.00
Simpson index	8	11	272	China: 6; Austria: 1; Catalan: 1	15.80–29.30	20.46–30.00
Sobs index	2	3	78	China: 2	25.10–26.90	n/a
Ace index	5	7	188	China: 4; Austria: 1	24.00–28.94	20.46–29.78
Coverage index	1	2	60	China: 1	25.10–26.90	n/a
Pielou’s evenness	2	2	99	Poland: 1; Catalan: 1	15.80–27.40	25.00–25.60
Amplicon sequence variants	2	3	169	Poland: 1; Catalan: 1	15.80–27.40	25.00–25.60
PD whole tree	8	10	228	China: 4; Austria: 2; Turkey: 1; Catalan: 1	15.80–29.30	20.46–30.00

*Abbreviations*: *PCOS* Polycystic ovary syndrome, *OTUs* Operational taxonomic units, *Ace* Abundance coverage estimator, *PD* Phylogenetic diversity, *BMI* Body mass index, *n/a* Not mentioned

The risk of bias in each included study was shown in Additional file 1: Supplemental Table 1. Eight high-risk studies were judged according to the Joanna Briggs Institute Critical Appraisal Checklist for Case-Control Studies [19, 26, 29, 36, 37, 40, 42, 43]. The most common shortcomings were the failure to report confounding factors identifying and strategies to deal with confounding factors.

### Synthesis of richness and diversity indices of alpha-diversity

Of the 26 studies, 984 patients and 888 controls were included in the meta-analyses (Additional file 1: Supplemental Table 2). Measurements of alpha-diversity included estimates of community richness (observed operational taxonomic units {OTUs}, observed species, Chao1 index, abundance coverage estimator {Ace} index, Sobs index, abundance coverage estimator), diversity/evenness (Shannon, Simpson, Pielou’s evenness), biodiversity (Faith phylogenetic diversity), and amplicon sequence variants. Original quantitative data of alpha-diversity indices are shown in Appendix file 1. The observed OTUs, observed species, Chao1, Shannon, Simpson, and PD indices in particular were widely used in the studies. As many indices were all calculated dependent on observed OTUs, we chose to assess richness using observed species and Chao1, and diversity through Shannon, Simpson, and PD indices.

**Fig. 2:**
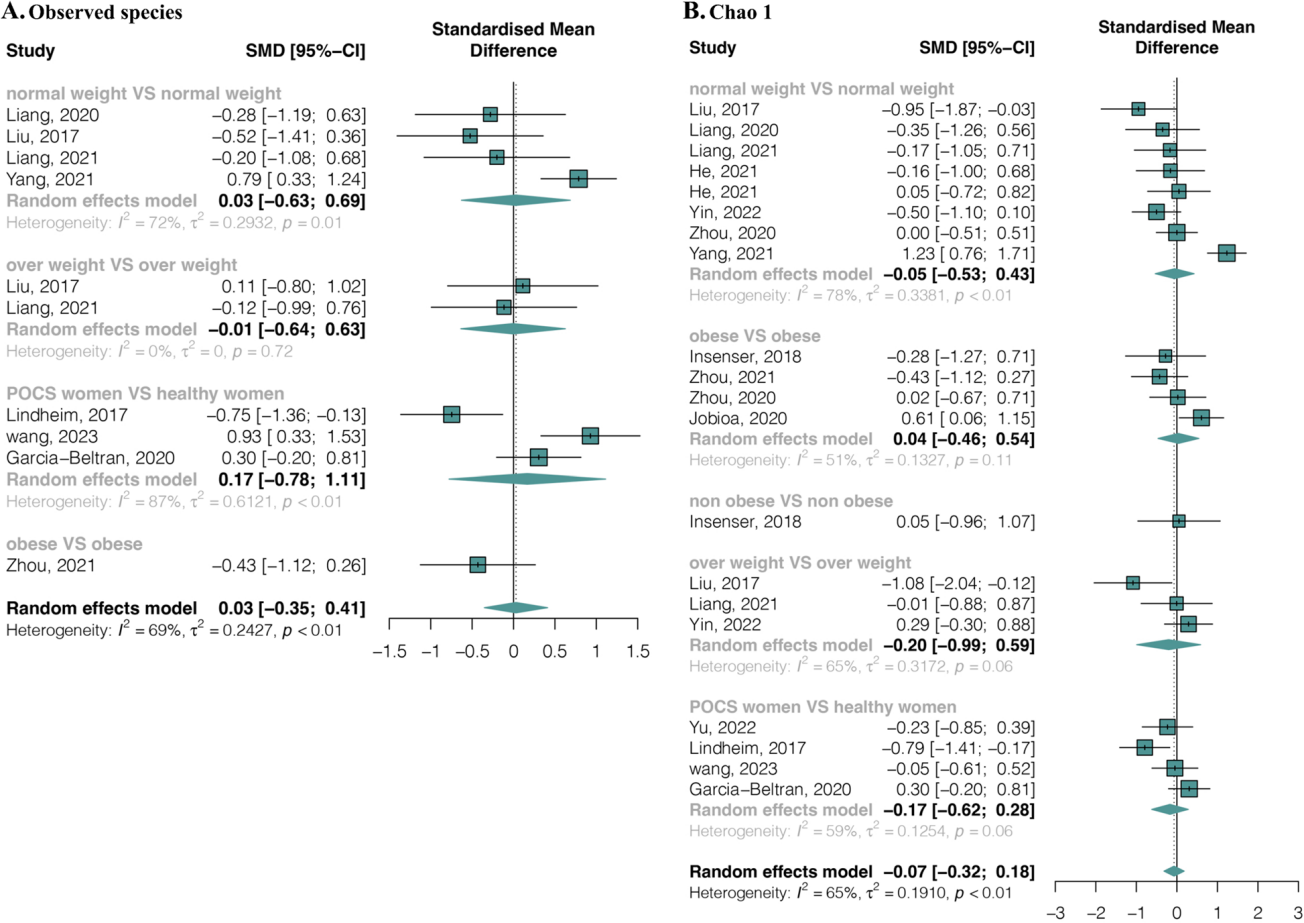
Alpha-diversity richness forest plots of PCOS compared with healthy controls in total and divided subgroups. **A** Observed species. **B** Chao1

Regarding richness, eight studies with ten estimates provided data on observed species in 214 PCOS and 165 healthy females. The pooled estimate showed high heterogeneity (*I*^2^ = 69.00%, *p* < 0.01) and no significant difference between groups (SMD = 0.03, 95% CI, − 0.35 to 0.41) (Fig. 2A). Similarly, BMI sub-grouping categories also showed no significant difference (Fig. 2A). Twelve studies with 20 estimates provided data on Chao1 in 400 PCOS and 327 healthy females, and no significant differences were observed between groups (SMD = − 0.07, 95% CI, − 0.32 to 0.18; *I*^2^ =65.00%, *p* < 0.01) nor within BMI sub-grouping categories (Fig. 2B). Publication bias was detected in the Chao 1 index by funnel plot (Additional file 1: Supplemental Figure 1) and Egger’s regression test (*t* = − 2.80, *p* = 0.01) (Additional file 1: Supplemental Table 4). Nevertheless, a stable result with no significant difference was observed by trim-and-fill analysis (Additional file 1: Supplemental Figure 2).

**Fig. 3:**
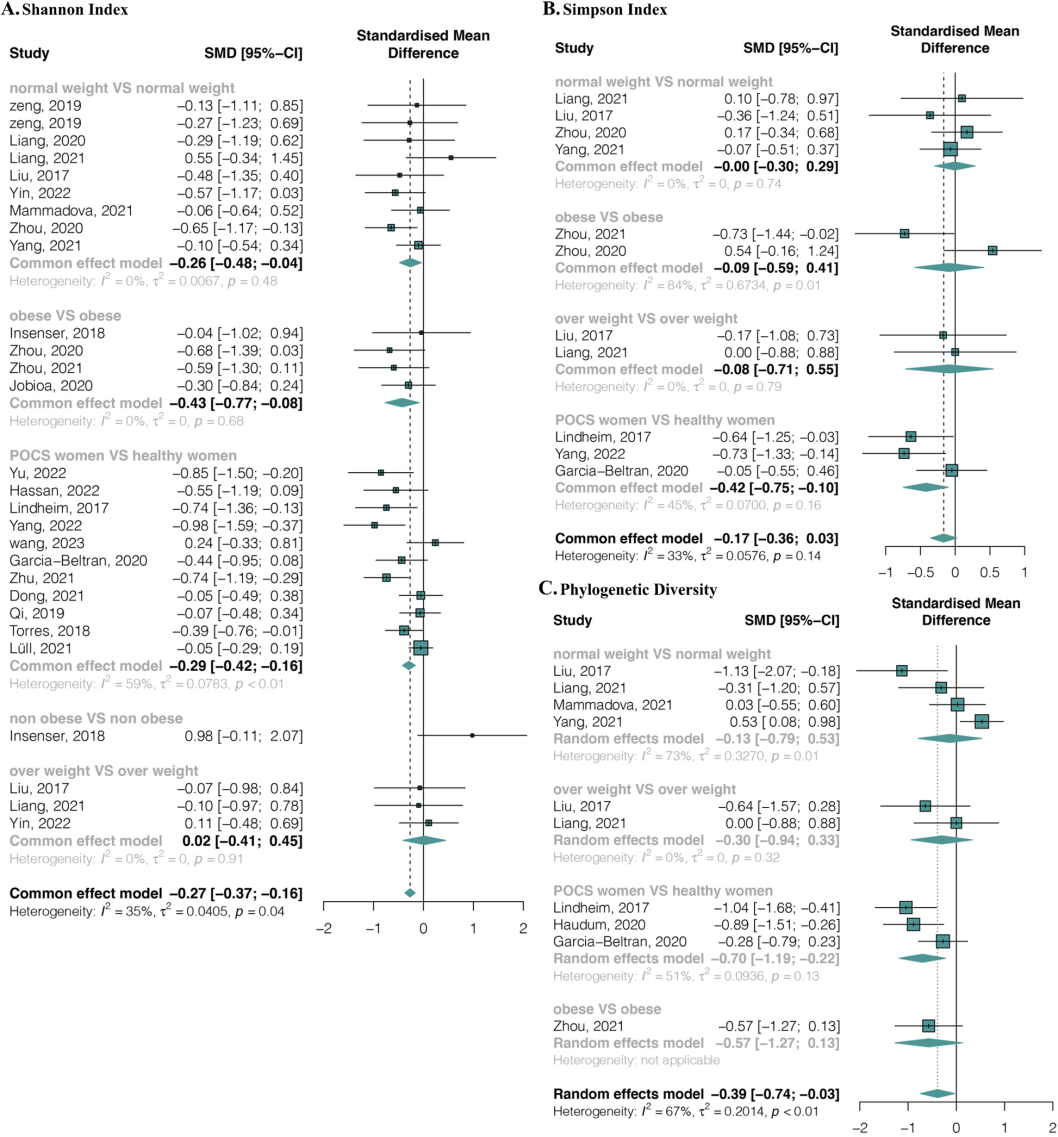
Alpha-diversity richness forest plots of PCOS compared with healthy controls in total and divided subgroups. **A** Shannon index. **B** Simpson index. **C** Phylogenetic diversity

As for diversity, 22 studies with 32 estimates provided data on the Shannon index in 896 PCOS and 806 healthy females. The pooled estimate demonstrated a significant decrease in PCOS patients compared with healthy females with a small effect size (SMD = − 0.27, 95% CI, − 0.37 to − 0.16) and no significant heterogeneity (*I*^2^ = 35.00%, *p* = 0.04) (Fig. 3A). Within the BMI sub-grouping categories, a significant decrease was only found between obese PCOS and obese healthy females (SMD = − 0.43, 95% CI, − 0.77 to -0.08; *I*^2^ = 0%, *p* = 0.68) (Fig. 3A). Simpson index data were provided in 8 studies with 11 estimates (272 PCOS and 191 control females). No significant difference was found between groups (SMD = − 0.17, 95% CI, − 0.36 to 0.03; *I*^2^ = 33.00%, *p* = 0.14) nor between any BMI sub-grouping categories (Fig. 3B). Finally, eight studies with ten estimates (228 PCOS and 174 healthy controls) provided phylogenetic diversity data. The pooled estimate showed a significant decrease in patients with a small effect size (SMD = − 0.39, 95% CI, − 0.74 to − 0.03) and significant heterogeneity (*I*^2^ = 67.00%, *p* < 0.01) (Fig. 3C). No publication biases were found in Shannon, Simpson, and PD indices (Additional file 1: Supplemental Figure 3, Supplemental Table 4).

Subgroup analyses and meta-regressions were performed to explore the sources of interstudy heterogeneity (observed species, Chao 1, phylogenetic diversity). Study areas, differences in age, BMI, total T, LH, FSH, LH/FSH, and HOMA-IR between PCOS and healthy control groups did not show any significant associations (Additional file 1: Supplemental Tables 5-8). In addition, sensitivity analyses were performed by removing low-quality studies and re-analyzed from the original sequence data. All alpha-diversity indices were stable except PD whole tree, which reported a significant decrease in PCOS patients compared to healthy controls after removing high-risk studies (SMD = − 0.50, 95% CI, − 0.73 to − 0.27; *I*^2^ = 30%, *p* < 0.01) (Additional file 1: Supplemental Table 8).

### Synthesis of beta-diversity

Beta-diversity comparison between PCOS patients and healthy controls was reported in 27 studies, using a variety of measures (Additional file 1: Supplemental Table 9) with most studies not grouping participants by BMI (20/27, 74.07%), while 7 studies grouped participants as “normal-weight,” “over-weight,” “obese,” and “non-obese.” Two studies reported a comparison between PCOS patients, with or without insulin resistance, and healthy females.

Although 20 out of 27 studies (74.07%) incorporated strategies to deal with confounding factors, primarily age and BMI (Additional file 1: Supplemental Table 1), inconsistent beta-diversities were reported, with nine studies reporting significant differences and 18 studies reporting non-significant differences. This variability is reiterated by one study reporting a significant difference between normal-weight PCOS patients and healthy controls, but a non-significant result between over-weight PCOS and healthy controls [18]. As various factors may affect beta-diversity findings, regression analysis was utilized to explore potential confounding factors such as study characteristics, endocrine parameters, and metabolic parameters. Our univariate logistic-regression analysis results did not reveal any significant confounding factors (Additional file 1: Supplemental Table 10).

### Synthesis of abundant microbial taxa differentially

**Fig. 4:**
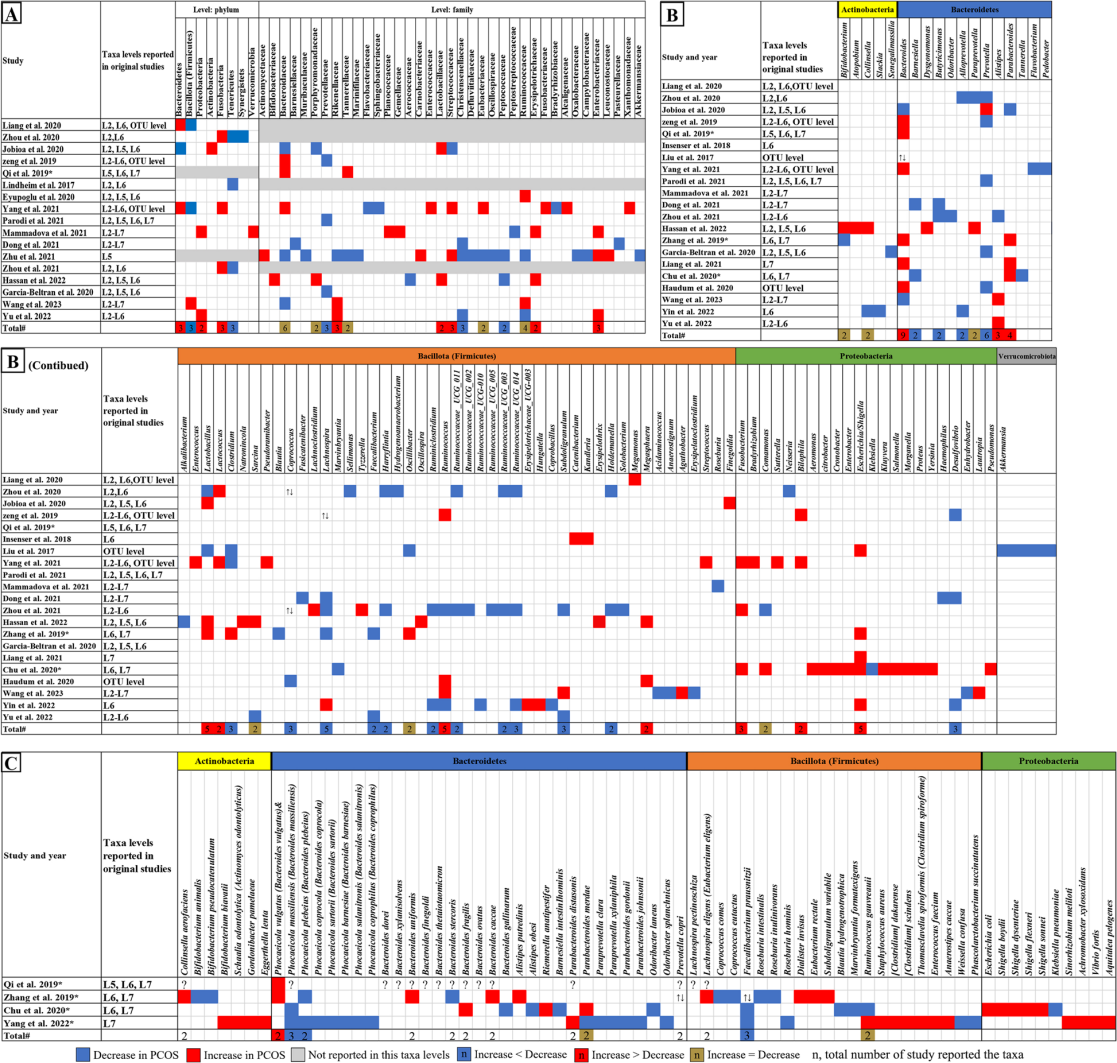
Changes in the relative abundance of microbes in the included studies. **A** Phylum and family level. **B** Genus level. **C** Species level. The red and blue grids indicate a statistically significant increase and decrease, respectively, in the taxa among patients with PCOS. The grey grids represent that the original studies did not report the results at the level of this taxa. In the total row, the numerical value in the grid indicates the count of studies that have reported a statistically significant alteration in the taxa. The red and blue grids represent the number of studies that have reported a statistically significant increase and decrease with PCOS, while the brown grids represent the equivalent number of studies that have reported a statistically significant increase as opposed to those reported a decrease. * represents the study with shotgun metagenomic sequencing. ↑↓ represents that there were increased and decreased species or OTU belonging to the taxa reported in studies. ? represents that the taxa were reported significantly changed without mentioning increasing or decreasing in PCOS. L2 to L7 represent phylum, class, order, family, genus, and species level respectively. OTUs, operational taxonomic units

Twenty-eight studies reported the relative abundance of gut microbes in PCOS patients versus healthy controls at various levels. Differences spanning 8 phyla, 37 families, and 88 genera were observed. We summarized the comparison of the relative abundance of bacteria in each study and the qualitative synthesis of overlapping results in Fig. 4.

Despite the high heterogeneity of gut microbes across studies, we observed significantly increased Fusobacteria, Proteobacteria, and Bacteroidetes at the phylum level, Streptococcaceae, Rikenellaceae, Lactobacillaceae, Erysipelotrichaceae, and Enterobacteriaceae at the family level, and *Bacteroides*, *Parabacteroides*, *Lactobacillus*, *Lactococcus*, *Megamonas*, *Fusobacterium*, *Bilophila*, and *Escherichia*/*Shigella* at the genus level in PCOS patients when compared to healthy controls. In contrast, there were significant reductions in Tenericutes and Firmicutes at the phylum level, Prevotellaceae, Christensenellaceae, and Peptococcaceae at the family level, and *Alloprevotella*, *Coprococcus*, *Ruminiclostridium*, *Faecalibacterium*, *Barnesiella*, *Butyricimonas*, *Prevotella*, *Clostridium*, *Desulfovibrio*, *Lachnospira*, *Harryflintia*, *Ruminococcaceae_UCG_003*, *Ruminococcaceae_UCG_014*, *Subdoligranulum*, and *Holdemanella* at the genus level. Notably, the genera with strong supporting evidence of an increase in PCOS cohorts included *Fusobacterium* (three studies), *Escherichia*/*Shigella* (five studies), *Lachnospira* (four studies), *Lactobacillus* (three studies reported a significant increase, two studies reported a significant decrease), *Prevotella* (five studies reported a significant decrease, one study reported a significant increase), and *Bacteroides* (seven studies reported a significant increase, three studies reported a significant decrease). In addition, Fig. 4C shows that four studies reported species abundance of gut microbes using shotgun metagenomic sequencing, with species *Bacteroides vulgatus* was significantly increase and *Faecalibacterium prausnitzii*, *Bacteroides massiliensis*, and *Bacteroides plebeius* were significantly decreased in two or more studies.

### Synthesis of bacterial function

Among the 28 included studies, there were four studies [5, 22, 23, 40] (Table 2, Additional file 1: Supplemental Table 11) that assessed the bacterial function through gut metagenomic sequencing. For those pathways consistently observed in two or more studies, the PCOS-associated pathways included folate biosynthesis, glycerophospholipid metabolism, biotin metabolism, cationic antimicrobial peptide resistance, lipopolysaccharide biosynthesis, phosphotransferase system, and fatty acid biosynthesis.

**Table 2:** Summaries of the bacterial function description from the included metagenomic studies

**Study**	**Bacterial function**^a^
Qi, et al. 2019 [5]	Folate biosynthesis; Glycerophospholipid metabolism; Biotin metabolism;
Zhang, et al. 2019 [22]	Folate biosynthesis; cationic antimicrobial peptide resistance; lipopolysaccharide biosynthesis; phosphotransferase system; fatty acid biosynthesis;
Chu, et al. 2020 [23]	Glycerophospholipid metabolism; cationic antimicrobial peptide resistance; lipopolysaccharide biosynthesis; phosphotransferase system;
Yang, et al. 2022 [40]	Biotin metabolism; fatty acid biosynthesis;

^a^All the bacterial functions described in two or more studies were included

Furthermore, while one study employed targeted gut metabolomics to investigate specific bile acid metabolites [5], another study employed untargeted metabolomics to assess the entire gut metabolome [28]. The study utilizing targeted gut metabolomics found substantial reductions in glycodeoxycholic acid and tauroursodeoxycholic acid in the PCOS group compared to the control group. In contrast, the study using untargeted metabolomics observed increases in arachidonic acid, taurocholic acid, 8,11,14-eicosatrienoic acid, docosahexaenoic acid, DHEA sulfate, and adrenic acid in the PCOS group, while testosterone was decreased. Moreover, two studies demonstrated that the administration of glycodeoxycholic acid [5] and chenodeoxycholic acid [37] could improve PCOS phenotypes in animal experiments respectively.

## Discussion

This systematic review firstly assesses the gut microbiota dysbiosis in PCOS. We observed consistency in the pattern of gut microbial dysbiosis despite variations across studies, which suggests an important role of gut microbiota in PCOS. The main findings of this study include the following: (1) the alterations in alpha-diversity indices indicated the disruption of bacterial phylogenetic abundance and ecological evenness while the observed bacteria showed only a mild change; (2) although the compositional changes of gut microbiota had been reported in many studies, there was inconsistency in the reporting of beta-diversity across studies; (3) dysbiosis including depletion of SCFA-related beneficial bacteria and bile-acid-metabolizing bacteria and enrichment of pro-inflammatory bacteria were observed in PCOS patients.

A robust microbial diversity is essential to the gut microbiota’s resilience to stress and is a key indicator of good health. Reduced diversity may result from the enrichment of pathogenic microorganisms and indicates a less healthy state in general [44]. In concordance with previous studies [45], our meta-analysis also showed decreased Shannon index and phylogenetic diversity index in PCOS patients. For other measurements, there were variances across all studies, with overall no statistically significant results when the Chao1 index, Simpson index, and observed species were assessed. It is important to highlight that different alpha diversity indices indicate different microbial profiles. For instance, while the Chao 1 index and the observed species are based on the total number of bacteria within a community, the Shannon index and the phylogenetic diversity index additionally consider bacterial evenness and phylogenetic abundance, respectively. Therefore, though there was heterogeneity between the studies, including differences in region, ethnicity, and methodologies, the statistically significant difference between alpha-diversity indices suggests that dysbiosis was manifested through the reduction of phylogenetic abundance and disruption of bacterial evenness rather than a change in the number of bacteria. As pointed out by Shade [44], the common assumption that “higher diversity is better” oversimplifies complex mechanisms, and understanding the mechanism behind these diversity indicators with contextual data would advance knowledge. Our analysis of alpha-diversity suggests that the dysbiotic microbial profile in PCOS patients would be the result of disproportional bacterial taxa rather than the changes of certain bacterial presence. These results indicated that the PCOS-related gut microbial profile might be featured by the changes of species within genera, which is out of the resolution of 16S sequencing and requires metagenomic sequencing to further evaluate and identify disease-specific biomarkers. Moreover, it is worth pointing out that the interstudy heterogeneity of alpha-diversity in pooled estimation decreased in certain subgroups; for instance, the heterogeneity of the Shannon index was lower within the normal, overweight, or obese group. To some extent, we also noted a decrease in other indices. This observation echoed the previous findings [46] that metabolic dysfunction could be an important factor influencing both gut microbiota and PCOS. Similarly, for our analysis on beta-diversity, significantly inconsistent results were observed between 24 included studies with no potential confounding factors revealed by logistic regression analysis. A common phenomenon is that most studies do not conduct subgroup analysis according to the characteristics of samples when analyzing beta-diversity, which limits the discovery of confounding factors that affect beta-diversity. Nevertheless, further exploration is needed to confirm the link between alpha-diversity, beta-diversity, PCOS patients, and healthy controls.

There is evidence that gut microbial dysbiosis could play a causal role in PCOS [5]. This provides a novel angle for researchers to propose gut bacteria as a potential etiology of PCOS. It has also been reported that PCOS patients exhibit increased pro-inflammatory state [47], disrupted gut barrier [48], and metabolic disorders [49], all of which are also considered to be the result of interactions between the gut microbiota and the host [50, 51]. In this way, a connection between the gut microbiota and PCOS could be established. In the present study, despite the variations and complexity of the gut microbiota, we observed several distinct microbial signatures in PCOS patients. In patients with PCOS, there were enrichment of *Fusobacterium*, *Escherichia*, and *Bacteroides* at the genus level and *Bacteroides fragilis* and *Escherichia coli* at the species level. Interestingly, *Fusobacterium* has also been observed to be enriched in the oral microbiota of PCOS patients [52]. *Fusobacterium* is known to be an opportunistic pathogen and its pathogenic role has been suggested in a variety of diseases [53, 54]. An abundance of *Fusobacterium* is involved in promoting inflammation and increasing gut barrier permeability in metabolic disorders [55]. These detrimental characteristics echo the manifestations of inflammation and metabolic disorders in PCOS [49]. Similarly, *Escherichia* could increase the virulence of commensal bacteria by enhancing mucosal attachment, invasion, and intracellular persistence, which leads to epithelial dysfunction and increased barrier permeability [56]. The biological mechanism that underpins the role of the gut microbiota and its metabolites in linking gut permeability and PCOS has been previously postulated [57]. *Bacteroides* are usually commensal in the gut, but some strains of *Bacteroides* have been identified as opportunistic pathogens [58]. The enterotoxigenic *Bacteroides fragilis*, which has the toxic gene encoding fragilysin, has been associated with different diseases [59, 60]. *Bacteroides vulgatus* has also been shown to be enriched in the gut microbiota of PCOS patients and induced PCOS-like symptoms in a murine model by altering bile acid metabolism and host immune response [5], which is the only differential species whose effect on PCOS was validated in animal model. Other consistent microbes also worth to be investigated in further study.

Based on the bacterial taxonomy and function results, our findings indicated several patterns of microbial disorder in PCOS. The clusters of bacteria that harbor beneficial or adverse effects to the host were identified, though single bacteria were weakly reproduced among studies. Microbial patterns that predominated in PCOS were of SCFA-producing and bile-acid-metabolizing bacteria, which was also evidenced by the altered bacterial function of secondary bile acid biosynthesis [5] and fatty acid biosynthesis [22, 40].

Among the included studies, several SCFA-producing bacteria were consistently reported depleted in PCOS, including *Butyricimonas*, *Blautia*, *Coprococcus*, and *Faecalibacterium prausnitzii*. These bacteria are regarded as SCFA producers and have beneficial effects on hosts, which were discussed in the included studies. Specifically, the SCFA producers were identified in the 16S sequencing studies, which was further supported by the observation of the bacterial function of SCFA metabolism in gut metagenomics. SCFAs, mainly acetate, propionate, and butyrate, are generated by gut bacteria metabolizing dietary elements and prebiotics [61]. Studies have shown that SCFAs play an important role in mitigating inflammation and maintaining gut barrier function through the stimulation, synthesis, and release of phagocytic molecules, anti-inflammatory cytokines, chemokines, and protective peptides [62, 63]. Butylated starch could be metabolized by the gut microbiota to produce SCFAs, which could stimulate the peptide-tyrosine-tyrosine secretion and the hypothalamic-pituitary-ovarian axis to alleviate PCOS [64]. In parallel with these research findings, it has been demonstrated that *F. prausnitzii* has anti-inflammatory capacities and supports mucosal immune homeostasis [65, 66], making it a next-generation probiotic with great therapeutic potential. One mechanism for these beneficial effects may be mediated by its ability to produce butyrate [67]. Butyrate can reduce oxidative stress and pro-inflammatory activity by maintaining the integrity of the gut barrier and limiting the translocation of bacteria and bacterial components such as lipopolysaccharide into the systemic circulation [68, 69]. In a recent study, Zhang and colleagues [22] further demonstrated that the colonization of *Bifidobacterium lactis*, an SCFA-producing probiotic, was related to the fluctuations in the levels of SCFAs, sex hormones, and signal peptides, which proposed a potential mechanism of how probiotics interact and regulate with the host. However, there was no report on SCFAs employing gut metabolomics in the included studies. Thus, the SCFAs profile in the gut would be of interest in future studies.

Bacteria involved in bile acid metabolism have also been associated with PCOS. Bile acids are produced in the liver by the oxidation of cholesterol which is catalyzed by a series of cytochromes P450 [70]. After a meal, bile acids are released into the duodenum where it is conjugated, and then the conjugated bile acids are reabsorbed from the ileum to the liver through the portal vein [71]. This cycle preserves more than 95% of the bile acid pool [71]. A small proportion of bile acids are secreted into the colon where they are mainly bio-transformed by the gut microbiota or excreted into faces [71, 72]. Most gram-positive bacteria, like *Ruminococcus* and *Clostridium*, and some gram-negative bacteria can metabolize bile acids [73]. Indeed, Qi and colleagues reported that the alteration of gut microbiota was associated with the reduction of bile acid (glycodeoxycholic acid and tauroursodeoxycholic acid) and IL-22 secretion [5]. Moreover, Yang and colleagues demonstrated that administration of chenodeoxycholic acid could improve PCOS phenotypes in mice experiments. Since bile acids play a crucial role in food digestion and energy metabolism, dysfunction in the secretion and reabsorption of bile acids could be a characteristic of insulin resistance, obesity, and type 2 diabetes [74, 75]. Additionally, bile acid receptors, Farnesoid X receptor and G-protein coupled bile acid receptor 1, regulate various elements of glucose, lipid, and energy metabolism [74]. While metabolic disorders, including hyperinsulinemia, insulin resistance and obesity, co-occur with PCOS [49], further studies revealing the relationship between bile acid and PCOS are needed to determine the etiological role of gut microbiota-mediated regulation of bile acids in PCOS.

It is noteworthy that the estrobolome, which is defined as “the aggregate of enteric bacterial genes whose products are capable of metabolizing estrogens” by Plottel and Blaser [76], has also been closely related to gynecological diseases [73, 77, 78]. In a review by Kwa and colleagues [79], 60 bacterial genera from human gut microbiota have the potential to encode β-glucuronidase and/or β-galactosidase using the data from Human Microbiome Project. This includes *Bacteroides*, *Bifidobacterium*, *Escherichia*, *Faecalibacterium*, and *Lactobacillus*, which were altered in PCOS patients across studies within this meta-analysis and postulated to be capable of impacting endogenous estrogen metabolism [76]. The enriched bacterial hydroxysteroid deconjugate activity may contribute to the modulation of the interconversion of conjugated forms of estrogens as well as androgenic molecules [80], which could also play a role in hormonal dysregulation in PCOS. The astrobleme is under-studied in the context of PCOS, and therefore, further research on the astrobleme could provide novel insights into understanding the role of the gut microbiota in hormone-mediated conditions like PCOS.

We performed a comprehensive meta-analysis by examining 28 papers, with a combined total of 1022 patients, and demonstrated the impact of the gut microbiota on PCOS. Our findings support dysbiosis as a hypothesized cause of PCOS, with disease-specific changes in microbial composition. However, there were several limitations of this study: (1) the sample sizes of current studies on the role of gut microbiota in PCOS were relatively limited, and thus, our meta-analysis might be underpowered and future studies are required to validate our findings in a larger population; (2) though microbial patterns were observed, disease-specific bacteria varied between studies. This could be in part due to the heterogeneity of the gut microbiota between study cohorts and their complex interactions with physiological and environmental factors. In addition, differences in methodology, including wet and dry lab protocols, would widen this gap; (3) the studies included in our meta-analysis were mostly based on 16S rRNA gene sequencing, which limited the interpretation at a species level. Furthermore, sequencing datasets were rarely provided, which rendered it difficult for researchers to integrate data and perform bioinformatic analyses using the same pipeline; (4) significant heterogeneity was observed in the pooled analysis of Chao 1, observed species, and PD whole tree indices. Although we performed sub-group and meta-regression analyses, no confounding factors were discovered that might elucidate the source of heterogeneity; (5) publication bias was observed in studies that reported Chao 1, but trim-and-fill analysis revealed that our result was stable; (6) most of the studies did not discern between patients that were newly diagnosed or currently under therapies. Further studies should address this point as the type of medication provided is a major factor that could profoundly influence the gut microbiota.

## Conclusions

This is the first meta-analysis to link gut microbial dysbiosis with PCOS. The evenness and phylogenetic abundance of gut microbiota in patients with PCOS was decreased when compared to healthy controls, while the diversity indices were overall preserved. Alterations of specific bacteria and clusters of bacteria indicated a link between gut-hormone-immunity crosstalk. Together, these characteristics in the gut microbiota of PCOS patients provide evidence for further exploration of the etiology of PCOS and potential novel therapeutic targets.

Our findings extend the results from previous studies that suggest gut dysbiosis in patients with PCOS is characterized by a shift in microbial balance to favor pro-inflammatory rather than anti-inflammatory bacteria. It also reiterates that studies should be interpreted with caution due to the inherent heterogeneity in the gut microbiota composition between different individuals.

### Supplementary Information

**Additional file 1:** **Supplemental Table 1.** [Quality assessment of the included original studies]. **Supplemental Table 2.**[Detailed characteristics of the included studies measured alpha diversity indexes]. **Supplemental Table 3.** [Stool sample processing methods in the included studies]. **Supplemental Table 4.** [Publish bias assessment by egger regression test in alpha diversity indexes]. **Supplemental Table 5.** [Subgroup and meta-regression analysis of α-diversity (Chao 1) difference between PCOS patients and healthy control]. **Supplemental Table 6.** [Subgroup and meta-regression analysis of α-diversity (Observed species) difference between PCOS patients and healthy control]. **Supplemental Table 7.** [Subgroup and meta-regression analysis of α-diversity (PD) difference between PCOS patients and healthy control]. **Supplemental Table 8.** [Sensitivity analyses of α-diversity indices difference between PCOS patients and healthy control]. **Supplemental Table 9.** [Summary of various measures of beta-diversity]. **Supplemental Table 10.** [Univariate logistic-regression analysis of factors potentially associated with Beta-diversity]. **Supplemental Table 11.** [Summaries of the bacterial function description from the included studies]. **Supplemental Figure 1.** [Funnel plot (A) and trim-and-fill funnel plot (B) assessing publication bias in themeta-analyses of Chao 1 index]. **Supplemental Figure 2.** [Forest plot by trim-and-fill analysis of Chao 1]. **Supplemental Figure 3.** [Funnel plot of assessing publication bias in the meta-analyses of observed species (A), Shannon (B), Simpson (C), and PD whole tree indexes (D)].

**Additional file 2:** **SupplementalTable 12.** [the clinical characteristics and sequencinginformation of the studies reported alpha diversity].

## Data Availability

All data generated or analyzed during this study are included in this published article and its supplementary information files.

## Abbreviations

PCOS: Polycystic ovary syndrome
SMD: Standardized mean difference
CIs: Confidence intervals
SCFA: Short-chain fatty acid
PRISMA: Preferred Reporting Items for Systematic Reviews and Meta-Analyses
BMI: Body mass index
Total T: Total testosterone
LH: Luteinizing hormone
FSH: Follicle-stimulating hormone
HOMA-IR: Homeostatic model assessment for insulin resistance
M: Means
SD: Standard deviations
ASVs: Amplicon sequence variants
OTUs: Operational taxonomic units
Ace: Abundance coverage estimator
PD: Phylogenetic diversity
