# Supplementary material for: Perturbations in gut microbiota composition in patients with polycystic ovary syndrome: a systematic review and meta-analysis
Source: BMC Med. 2023 Aug 9;21:302. doi: 10.1186/s12916-023-02975-8 (PMC10413517; doi:10.1186/s12916-023-02975-8)
Supplement: Supplementary file 1 — Additional file 1: Supplemental Table 1. [Quality assessment of the included original studies]. Supplemental Table 2.[Detailed characteristics of the included studies measured alpha diversity indexes]. Supplemental Table 3. [Stool sample processing methods in the included studies]. Supplemental Table 4. [Publish bias assessment by egger regression test in alpha diversity indexes]. Supplemental Table 5. [Subgroup and meta-regression analysis of α-diversity (Chao 1) difference between PCOS patients and healthy control]. Supplemental Table 6. [Subgroup and meta-regression analysis of α-diversity (Observed species) difference between PCOS patients and healthy control]. Supplemental Table 7. [Subgroup and meta-regression analysis of α-diversity (PD) difference between PCOS patients and healthy control]. Supplemental Table 8. [Sensitivity analyses of α-diversity indices difference between PCOS patients and healthy control]. Supplemental Table 9. [Summary of various measures of beta-diversity]. Supplemental Table 10. [Univariate logistic-regression analysis of factors potentially associated with Beta-diversity]. Supplemental Table 11. [Summaries of the bacterial function description from the included studies]. Supplemental Figure 1. [Funnel plot (A) and trim-and-fill funnel plot (B) assessing publication bias in themeta-analyses of Chao 1 index]. Supplemental Figure 2. [Forest plot by trim-and-fill analysis of Chao 1]. Supplemental Figure 3. [Funnel plot of assessing publication bias in the meta-analyses of observed species (A), Shannon (B), Simpson (C), and PD whole tree indexes (D)]. [file 12916_2023_2975_MOESM1_ESM.docx]

**PCOS_Meta Supplemental materials**

**Appendix 1. Electronic search strategy in PubMed, Embase and Cochrane Library** **databases**

**Databases Search deadline：2023/5/22**

**PubMed**

| step | Search | **Hits** |
| --- | --- | --- |
| #1 | (((((gut[Title/Abstract]) OR (gastrointestinal[Title/Abstract])) OR (intestinal[Title/Abstract])) OR (feacal[Title/Abstract])) OR (fecal[Title/Abstract])) OR (stool[Title/Abstract]) AND (humans[Filter]) | **444928** |
| #2 | ((((((microbiome[Title/Abstract]) OR (microbiota[Title/Abstract])) OR (ecosystem[Title/Abstract])) OR (bacteria[Title/Abstract])) OR (flora[Title/Abstract])) OR (microflora[Title/Abstract])) OR (dysbiosis[Title/Abstract]) AND (humans[Filter]) | **221836** |
| #3 | #1 AND #2 | **57550** |
| #4 | (((((((((((((((Ovary Syndrome, Polycystic[Title/Abstract]) OR (Syndrome, Polycystic Ovary[Title/Abstract])) OR (Stein-Leventhal Syndrome[Title/Abstract])) OR (Stein Leventhal Syndrome[Title/Abstract])) OR (Syndrome, Stein-Leventhal[Title/Abstract])) OR (Sclerocystic Ovarian Degeneration[Title/Abstract])) OR (Ovarian Degeneration, Sclerocystic[Title/Abstract])) OR (Sclerocystic Ovary Syndrome[Title/Abstract])) OR (Polycystic Ovarian Syndrome[Title/Abstract])) OR (Ovarian Syndrome, Polycystic[Title/Abstract])) OR (Polycystic Ovary Syndrome 1[Title/Abstract])) OR (Sclerocystic Ovaries[Title/Abstract])) OR (Ovary, Sclerocystic[Title/Abstract])) OR (Sclerocystic Ovary[Title/Abstract])) OR (hyperandrogenism[Title/Abstract])) OR (PCOS[Title/Abstract]) AND (humans[Filter]) | **15411** |
| #5 | #3 AND #4 | **105** |
| Limits: human, English | | |

**Embase AND Medline**

| step | Search | **Hits** |
| --- | --- | --- |
| #1 | gut:ab,ti OR gastrointestinal:ab,ti OR intestinal:ab,ti OR fecal:ab,ti OR feacal:ab,ti OR stool:ab,ti | **975050** |
| #2 | microbiome:ab,ti OR microbiota:ab,ti OR ecosystem:ab,ti OR bacteria:ab,ti OR flora:ab,ti OR microflora:ab,ti OR dysbiosis:ab,ti | **722838** |
| #3 | 'ovary syndrome, polycystic':ab,ti OR 'syndrome, polycystic ovary':ab,ti OR 'stein-leventhal syndrome':ab,ti OR 'stein leventhal syndrome':ab,ti OR 'syndrome, stein-leventhal':ab,ti OR 'sclerocystic ovarian degeneration':ab,ti OR 'ovarian degeneration, sclerocystic':ab,ti OR 'sclerocystic ovary syndrome':ab,ti OR 'polycystic ovarian syndrome':ab,ti OR 'ovarian syndrome, polycystic':ab,ti OR 'polycystic ovary syndrome 1':ab,ti OR 'sclerocystic ovaries':ab,ti OR 'ovary, sclerocystic':ab,ti OR 'sclerocystic ovary':ab,ti OR hyperandrogenism:ab,ti OR pcos:ab,ti | **27904** |
| #4 | #1 AND #2 | **142668** |
| #5 | #3 AND #4 | **209** |
| #6 | #5 AND [humans]/lim | **163** |

**Web of Science**

| step | Search | **Hits** |
| --- | --- | --- |
| #1 | (((((AB=(gut)) OR AB=(gastrointestinal)) OR AB=(intestinal)) OR AB=(fecal)) OR AB=(feacal)) OR AB=(stool) | **957172** |
| #2 | ((((((AB=(microbiome)) OR AB=(microbiota)) OR AB=(ecosystem)) OR AB=(bacteria)) OR AB=(flora)) OR AB=(microflora)) OR AB=(dysbiosis) | **1514971** |
| #3 | (((((((((((((((AB=(ovary syndrome, polycystic)) OR AB=(syndrome, polycystic ovary)) OR AB=(stein-leventhal syndrome)) OR AB=(stein leventhal syndrome)) OR AB=(syndrome, stein-leventhal)) OR AB=(sclerocystic ovarian degeneration)) OR AB=(ovarian degeneration, sclerocystic)) OR AB=(sclerocystic ovary syndrome)) OR AB=(polycystic ovarian syndrom)) OR AB=(ovarian syndrome, polycystic)) OR AB=(polycystic ovary syndrome 1)) OR AB=(sclerocystic ovaries)) OR AB=(ovary, sclerocystic)) OR AB=(sclerocystic ovary)) OR AB=(hyperandrogenism)) OR AB=(pcos) | **25844** |
| #4 | #1 AND #2 | **152566** |
| #5 | #3 AND #4 | **218** |
| #6 | #3 AND #4 and Human (Search within topic) | **131** |

**Cochrane Library**

| step | Search | **Hits** |
| --- | --- | --- |
| #1 | (gut):ti,ab,kw OR (gastrointestinal):ti,ab,kw OR (intestinal):ti,ab,kw OR (fecal):ti,ab,kw OR (stool):ti,ab,kw | **87407** |
| #2 | (microbiome):ti,ab,kw OR (microbiota):ti,ab,kw OR (ecosystem):ti,ab,kw OR (bacteria):ti,ab,kw OR (flora):ti,ab,kw | **25307** |
| #3 | (microflora):ti,ab,kw OR (dysbiosis):ti,ab,kw | **3209** |
| #4 | #2 OR #3 | **26046** |
| #5 | (ovary syndrome, polycystic):ti,ab,kw OR (syndrome, polycystic ovary):ti,ab,kw OR (stein-leventhal syndrome):ti,ab,kw OR (stein leventhal syndrome):ti,ab,kw OR (syndrome, stein-leventhal):ti,ab,kw (Word variations have been searched) | **4155** |
| #6 | (sclerocystic ovarian degeneration):ti,ab,kw OR (ovarian degeneration, sclerocystic):ti,ab,kw OR (sclerocystic ovary syndrome):ti,ab,kw OR (polycystic ovarian syndrom):ti,ab,kw OR (syndrome, stein-leventhal):ti,ab,kw | **2100** |
| #7 | (polycystic ovary syndrome 1):ti,ab,kw OR (sclerocystic ovaries):ti,ab,kw OR (ovary, sclerocystic):ti,ab,kw OR (sclerocystic ovary):ti,ab,kw OR (hyperandrogenism):ti,ab,kw | **2243** |
| #3 | (PCOS):ti,ab,kw | **3723** |
| #4 | #5 OR #6 OR #7 OR #8 | **4782** |
| #5 | #1 AND #4 AND #9 | **31** |

**Wiley Online Library**

| step | Search | **Hits** |
| --- | --- | --- |
| #1 | "(gut) OR (gastrointestinal) OR (intestinal) OR (fecal) OR (stool)" in Abstract | **117470** |
| #2 | "(microbiome) OR (microbiota) OR (intestinal) OR (ecosystem) OR (bacteria) OR (flora) OR (microflora) OR (dysbiosis)" in Abstract | **241606** |
| #3 | "(ovary syndrome, polycystic) OR (syndrome, polycystic ovary) OR (stein-leventhal syndrome) OR (stein leventhal syndrome) OR (syndrome, stein-leventhal) OR (Word variations have been searched) OR (sclerocystic ovarian degeneration) OR (ovarian degeneration, sclerocystic) OR (sclerocystic ovary syndrome) OR (polycystic ovarian syndrom) OR (syndrome, stein-leventhal) OR (polycystic ovary syndrome 1) OR (sclerocystic ovaries) OR (ovary, sclerocystic) OR (sclerocystic ovary) OR (hyperandrogenism) OR (PCOS)" in Abstract | **2664** |
| #4 | #1 AND #2 AND #3 | **23** |

Supplemental Table 1: Quality assessment of the included original studies.

| **Study and Year** | **1. Were the criteria for inclusion in the sample clearly defined?** | **2. Were the study subjects and the setting described in detail?** | **3. Was the exposure measured in a valid and reliable way?** | **4. Were objective, standard criteria used for measurement of the condition?** | **5. Were confounding factors identified?** | **6. Were strategies to deal with confounding factors stated?** | **7. Were the outcomes measured in a valid and reliable way?** | **8. Was appropriate statistical analysis used?** | **risk** |
| --- | --- | --- | --- | --- | --- | --- | --- | --- | --- |
| Liang, et al. 2020 | Y | Y | Y | Y | N | N | Y | Y | H |
| Zhou, et al. 2020 | Y | Y | Y | Y | Y | Y | Y | Y | L |
| Jobioa, et al. 2020 | Y | Y | Y | Y | N | N | Y | Y | H |
| zeng, et al. 2019 | Y | Y | Y | Y | Y | Y | Y | Y | L |
| Qi, et al. 2019 | Y | Y | Y | Y | Y | Y | Y | Y | L |
| Torres, et al. 2018 | Y | Y | Y | Y | Y | Y | Y | Y | L |
| Insenser, et al. 2018 | Y | Y | Y | Y | N | N | Y | Y | H |
| Liu, et al. 2017 | Y | Y | Y | Y | Y | Y | Y | Y | L |
| Lindheim, et al. 2017 | Y | Y | Y | Y | Y | Y | Y | Y | L |
| Eyupoglu, et al. 2020 | Y | Y | Y | Y | Y | Y | Y | Y | L |
| Yang et al. 2021 | Y | Y | Y | Y | N | N | Y | Y | H |
| Lüll et al. 2021 | Y | Y | Y | Y | Y | Y | Y | Y | L |
| Parodi et al. 2021 | Y | Y | Y | Y | N | N | Y | Y | H |
| Mammadova et al. 2021 | Y | Y | Y | Y | Y | Y | Y | Y | L |
| Dong et al. 2021 | Y | Y | Y | Y | Y | Y | Y | Y | L |
| Zhu et al. 2021 | Y | Y | Y | Y | Y | Y | Y | Y | L |
| Zhou et al. 2020 | Y | Y | Y | Y | Y | Y | Y | Y | L |
| Hassan et al. 2022 | Y | Y | Y | Y | Y | Y | Y | Y | L |
| He et al. 2021 | Y | Y | Y | Y | Y | Y | Y | Y | L |
| Zhang, et al. 2019 | Y | Y | Y | Y | Y | Y | Y | Y | L |
| Garcia-Beltran et al. 2020 | Y | Y | Y | Y | Y | Y | Y | Y | L |
| Liang et al. 2021 | Y | Y | Y | Y | Y | Y | Y | Y | L |
| Chu et al. 2020 | Y | Y | Y | Y | Y | Y | Y | Y | L |
| Haudum et al. 2020 | Y | Y | Y | Y | Y | Y | Y | Y | L |
| Wang et al. 2023 | Y | Y | Y | Y | N | N | Y | Y | H |
| Yin et al. 2022 | Y | Y | Y | Y | Y | Y | Y | Y | L |
| Yu et al. 2022 | Y | Y | Y | Y | N | N | Y | Y | H |
| Yang et al. 2022 | Y | Y | Y | Y | N | N | Y | Y | H |

Joanna Briggs Institute Critical Appraisal Checklist for Case Control Studies; Y, yes; N, no; H, high-risk; L, low-risk.

Supplemental Table 2. Detailed characteristics of the included studies measured alpha diversity indexes.

| **Study** | **year** | **analysis** | **country** | **Region** | **Specimen**  **type** | **Definite of PCOS** | **Study design** | **Microbiome assessment Method** | **measure** | **Case VS control** | **Case No.** | **Case age** | **Case BMI** | **Control NO.** | **Control age** | **Control BMI** |
| --- | --- | --- | --- | --- | --- | --- | --- | --- | --- | --- | --- | --- | --- | --- | --- | --- |
| Liang | 2020 | original | China | East | Fecal | Rotterdam criteria | Cross-sectional study | 16S rRNA, V3-4 | observed species | normal weight VS normal weight | 10 | 25.7 ± 3.5 | 20.7 | 9 | 27.9 ± 3.6 | 20.9 |
|  | 2020 | original | China | East | Fecal | Rotterdam criteria | Cross-sectional study | 16S rRNA, V3-4 | Shannon index | normal weight VS normal weight | 10 | 25.7 ± 3.5 | 20.7 | 9 | 27.9 ± 3.6 | 20.9 |
|  | 2020 | original | China | East | Fecal | Rotterdam criteria | Cross-sectional study | 16S rRNA, V3-4 | Chao1 | normal weight VS normal weight | 10 | 25.7 ± 3.5 | 20.7 | 9 | 27.9 ± 3.6 | 20.9 |
| Zhou | 2020 | original | China | East | Fecal | Rotterdam criteria | Cross-sectional study | 16S rRNA, V3-4 | Shannon index | normal weight VS normal weight | 30 | 25.1 ± 4.27 | NA | 30 | 22.1 ± 1.64 | NA |
|  | 2020 | original | China | East | Fecal | Rotterdam criteria | Cross-sectional study | 16S rRNA, V3-4 | Shannon index | obese VS obese | 30 | 26.9 ± 4.86 | NA | 11 | 25.3 ± 1.61 | NA |
|  | 2020 | original | China | East | Fecal | Rotterdam criteria | Cross-sectional study | 16S rRNA, V3-4 | Simpson Index | normal weight VS normal weight | 30 | 25.1 ± 4.27 | NA | 30 | 22.1 ± 1.64 | NA |
|  | 2020 | original | China | East | Fecal | Rotterdam criteria | Cross-sectional study | 16S rRNA, V3-4 | Simpson Index | obese VS obese | 30 | 26.9 ± 4.86 | NA | 11 | 25.3 ± 1.61 | NA |
|  | 2020 | original | China | East | Fecal | Rotterdam criteria | Cross-sectional study | 16S rRNA, V3-4 | Sobs index | normal weight VS normal weight | 30 | 25.1 ± 4.27 | NA | 30 | 22.1 ± 1.64 | NA |
|  | 2020 | original | China | East | Fecal | Rotterdam criteria | Cross-sectional study | 16S rRNA, V3-4 | Sobs index | obese VS obese | 30 | 26.9 ± 4.86 | NA | 11 | 25.3 ± 1.61 | NA |
|  | 2020 | original | China | East | Fecal | Rotterdam criteria | Cross-sectional study | 16S rRNA, V3-4 | Ace index | normal weight VS normal weight | 30 | 25.1 ± 4.27 | NA | 30 | 22.1 ± 1.64 | NA |
|  | 2020 | original | China | East | Fecal | Rotterdam criteria | Cross-sectional study | 16S rRNA, V3-4 | Ace index | obese VS obese | 30 | 26.9 ± 4.86 | NA | 11 | 25.3 ± 1.61 | NA |
|  | 2020 | original | China | East | Fecal | Rotterdam criteria | Cross-sectional study | 16S rRNA, V3-4 | Coverage index | normal weight VS normal weight | 30 | 25.1 ± 4.27 | NA | 30 | 22.1 ± 1.64 | NA |
|  | 2020 | original | China | East | Fecal | Rotterdam criteria | Cross-sectional study | 16S rRNA, V3-4 | Coverage index | obese VS obese | 30 | 26.9 ± 4.86 | NA | 11 | 25.3 ± 1.61 | NA |
|  | 2020 | original | China | East | Fecal | Rotterdam criteria | Cross-sectional study | 16S rRNA, V3-4 | Chao1 | normal weight VS normal weight | 30 | 25.1 ± 4.27 | NA | 30 | 22.1 ± 1.64 | NA |
|  | 2020 | original | China | East | Fecal | Rotterdam criteria | Cross-sectional study | 16S rRNA, V3-4 | Chao1 | obese VS obese | 30 | 26.9 ± 4.86 | NA | 11 | 25.3 ± 1.61 | NA |
| Jobioa | 2020 | original | America | West | Fecal | NIH | Prospective, case-control, cross-sectional study | 16S rRNA, V3-4 | Shannon index | obese VS obese | 37 | 16.1 ± 0.3 | 36 | 21 | 14.5 ± 0.4 | 35 |
|  | 2020 | original | America | West | Fecal | NIH | Prospective, case-control, cross-sectional study | 16S rRNA, V3-4 | Chao1 | obese VS obese | 37 | 16.1 ± 0.3 | 36 | 21 | 14.5 ± 0.4 | 35 |
| zeng | 2019 | original | China | East | Fecal | Rotterdam criteria | Pilot study | 16S rRNA, V3-4 | Shannon index | normal weight VS normal weight | 8 | 26.13 ± 7.06 | 22.58 | 8 | 26.38 ± 3.85 | 20.82 |
|  | 2019 | original | China | East | Fecal | Rotterdam criteria | Pilot study | 16S rRNA, V3-4 | Shannon index | normal weight VS normal weight | 9 | 25.11 ± 4.28 | 22.63 | 8 | 26.38 ± 3.85 | 20.82 |
|  | 2019 | original | China | East | Fecal | Rotterdam criteria | Pilot study | 16S rRNA, V3-4 | Observed OTUs | normal weight VS normal weight | 8 | 26.13 ± 7.06 | 22.58 | 8 | 26.38 ± 3.85 | 20.82 |
|  | 2019 | original | China | East | Fecal | Rotterdam criteria | Pilot study | 16S rRNA, V3-4 | Observed OTUs | normal weight VS normal weight | 9 | 25.11 ± 4.28 | 22.63 | 8 | 26.38 ± 3.85 | 20.82 |
| Qi | 2019 | original | China | East | Fecal | Rotterdam criteria | Cohort | METAGENOMICS | Shannon index | PCOS women VS healthy women | 50 | 29.9 ± 0.45 | 24.7 | 43 | 29.6 ± 0.56 | 23.7 |
| Torres | 2018 | original | Poland | West | Fecal | Rotterdam criteria | Cohort | 16S rRNA, V4 | Shannon index | PCOS women VS healthy women | 70 | 27.4 ± 4.9 | 25.6 | 47 | 29.4 ± 4.9 | 23.7 |
|  | 2018 | original | Poland | West | Fecal | Rotterdam criteria | Cohort | 16S rRNA, V4 | Pielou's Evenness | PCOS women VS healthy women | 70 | 27.4 ± 4.9 | 25.6 | 47 | 29.4 ± 4.9 | 23.7 |
|  | 2018 | original | Poland | West | Fecal | Rotterdam criteria | Cohort | 16S rRNA, V4 | amplicon sequence variants | PCOS women VS healthy women | 70 | 27.4 ± 4.9 | 25.6 | 47 | 29.4 ± 4.9 | 23.7 |
|  | 2018 | original | Poland | West | Fecal | Rotterdam criteria | Cohort | 16S rRNA, V4 | amplicon sequence variants | PCOS women VS healthy women | 70 | 27.4 ± 4.9 | 25.6 | 47 | 29.4 ± 4.9 | 23.7 |
| Insenser | 2018 | original | Spain | west | Fecal | Rotterdam criteria | Cross-sectional study | 16S rRNA, V4 | Shannon index | non obese VS non obese | 7 | 23.0 ± 7.9 | 24.4 | 8 | 27.3 ± 6.5 | 23.4 |
|  | 2018 | original | Spain | west | Fecal | Rotterdam criteria | Cross-sectional study | 16S rRNA, V4 | Shannon index | obese VS obese | 8 | 29.9 ± 5.1 | 37 | 8 | 27.3 ± 4.0 | 35.9 |
|  | 2018 | original | Spain | west | Fecal | Rotterdam criteria | Cross-sectional study | 16S rRNA, V4 | Chao1 | non obese VS non obese | 7 | 23.0 ± 7.9 | 24.4 | 8 | 27.3 ± 6.5 | 23.4 |
|  | 2018 | original | Spain | west | Fecal | Rotterdam criteria | Cross-sectional study | 16S rRNA, V4 | Chao1 | obese VS obese | 8 | 29.9 ± 5.1 | 37 | 8 | 27.3 ± 4.0 | 35.9 |
| Liu | 2017 | original | China | East | Fecal | Rotterdam criteria | Cross-sectional study | 16S rRNA, V3-4 | Chao1 | normal weight VS normal weight | 12 | 25.5 ± 4.3 | 21.6 | 9 | 32.2 ± 5.9 | 21.9 |
|  | 2017 | re-analysis | China | East | Fecal | Rotterdam criteria | Cross-sectional study | 16S rRNA, V3-4 | observed species | normal weight VS normal weight | 12 | 25.5 ± 4.3 | 21.6 | 9 | 32.2 ± 5.9 | 21.9 |
|  | 2017 | re-analysis | China | East | Fecal | Rotterdam criteria | Cross-sectional study | 16S rRNA, V3-4 | Shannon index | normal weight VS normal weight | 12 | 25.5 ± 4.3 | 21.6 | 9 | 32.2 ± 5.9 | 21.9 |
|  | 2017 | re-analysis | China | East | Fecal | Rotterdam criteria | Cross-sectional study | 16S rRNA, V3-4 | Simpson index | normal weight VS normal weight | 12 | 25.5 ± 4.3 | 21.6 | 9 | 32.2 ± 5.9 | 21.9 |
|  | 2017 | re-analysis | China | East | Fecal | Rotterdam criteria | Cross-sectional study | 16S rRNA, V3-4 | PD whole tree | normal weight VS normal weight | 12 | 25.5 ± 4.3 | 21.6 | 9 | 32.2 ± 5.9 | 21.9 |
|  | 2017 | re-analysis | China | East | Fecal | Rotterdam criteria | Cross-sectional study | 16S rRNA, V3-4 | observed species | overweight VS overweight | 21 | 29.3 ± 6.5 | 30 | 6 | 33 ± 5.4 | 27.5 |
|  | 2017 | re-analysis | China | East | Fecal | Rotterdam criteria | Cross-sectional study | 16S rRNA, V3-4 | Shannon index | overweight VS overweight | 21 | 29.3 ± 6.5 | 30 | 6 | 33 ± 5.4 | 27.5 |
|  | 2017 | re-analysis | China | East | Fecal | Rotterdam criteria | Cross-sectional study | 16S rRNA, V3-4 | Simpson index | overweight VS overweight | 21 | 29.3 ± 6.5 | 30 | 6 | 33 ± 5.4 | 27.5 |
|  | 2017 | re-analysis | China | East | Fecal | Rotterdam criteria | Cross-sectional study | 16S rRNA, V3-4 | PD whole tree | overweight VS overweight | 21 | 29.3 ± 6.5 | 30 | 6 | 33 ± 5.4 | 27.5 |
|  | 2017 | original | China | East | Fecal | Rotterdam criteria | Cross-sectional study | 16S rRNA, V3-4 | Observed OTUs | normal weight VS normal weight | 12 | 25.5 ± 4.3 | 21.6 | 9 | 32.2 ± 5.9 | 21.9 |
|  | 2017 | original | China | East | Fecal | Rotterdam criteria | Cross-sectional study | 16S rRNA, V3-4 | Observed OTUs | overweight VS overweight | 21 | 29.3 ± 6.5 | 30 | 6 | 33 ± 5.4 | 27.5 |
|  | 2017 | original | China | East | Fecal | Rotterdam criteria | Cross-sectional study | 16S rRNA, V3-4 | Chao1 | overweight VS overweight | 21 | 29.3 ± 6.5 | 30 | 6 | 33 ± 5.4 | 27.5 |
| Lindheim | 2017 | original | Austria | West | Fecal | Rotterdam criteria | Pilot cohort | 16S rRNA, V1-2 | Observed OTUs | PCOS women VS healthy women | 24 | median = 27, IQR = 5.9 | 24.9 | 20 | median = 32, IQR = 12.0 | 22.3 |
|  | 2017 | re-analysis | Austria | West | Fecal | Rotterdam criteria | Pilot cohort | 16S rRNA, V1-2 | Chao1 | PCOS women VS healthy women | 24 | median = 27, IQR = 5.9 | 24.9 | 20 | median = 32, IQR = 12.0 | 22.3 |
|  | 2017 | re-analysis | Austria | West | Fecal | Rotterdam criteria | Pilot cohort | 16S rRNA, V1-2 | Ace index | PCOS women VS healthy women | 24 | median = 27, IQR = 5.9 | 24.9 | 20 | median = 32, IQR = 12.0 | 22.3 |
|  | 2017 | re-analysis | Austria | West | Fecal | Rotterdam criteria | Pilot cohort | 16S rRNA, V1-2 | observed species | PCOS women VS healthy women | 24 | median = 27, IQR = 5.9 | 24.9 | 20 | median = 32, IQR = 12.0 | 22.3 |
|  | 2017 | re-analysis | Austria | West | Fecal | Rotterdam criteria | Pilot cohort | 16S rRNA, V1-2 | Shannon index | PCOS women VS healthy women | 24 | median = 27, IQR = 5.9 | 24.9 | 20 | median = 32, IQR = 12.0 | 22.3 |
|  | 2017 | re-analysis | Austria | West | Fecal | Rotterdam criteria | Pilot cohort | 16S rRNA, V1-2 | Simpson index | PCOS women VS healthy women | 24 | median = 27, IQR = 5.9 | 24.9 | 20 | median = 32, IQR = 12.0 | 22.3 |
|  | 2017 | original | Austria | West | Fecal | Rotterdam criteria | Pilot cohort | 16S rRNA, V1-2 | PD whole tree | PCOS women VS healthy women | 24 | median = 27, IQR = 5.9 | 24.9 | 20 | median = 32, IQR = 12.0 | 22.3 |
| Eyupoglu | 2020 | original | Turkey | West | Fecal | Rotterdam criteria | Cross-sectional study | 16S rRNA, V3-4 | Observed OTUs | PCOS women VS healthy women | 17 | 20 (IQR=19-22) | 29.6 | 15 | median = 22, IQR = 18-27 | 31.5 |
|  | 2020 | original | Turkey | West | Fecal | Rotterdam criteria | Cross-sectional study | 16S rRNA, V3-4 | Observed OTUs | overweight VS overweight | 10 | NA | NA | 8 | NA | NA |
|  | 2020 | original | Turkey | West | Fecal | Rotterdam criteria | Cross-sectional study | 16S rRNA, V3-4 | Observed OTUs | obese VS obese | 7 | NA | NA | 7 | NA | NA |
| Yang | 2021 | re-analysis | China | East | Fecal | Rotterdam criteria | Cross-sectional study | 16S rRNA, V4 | Observed OTUs | normal weight VS normal weight | 56 | 24.00 (22.00, 27.00) | 21.07 | 31 | 26.00 (24.00, 27.00) | 19.8 |
|  | 2021 | original | China | East | Fecal | Rotterdam criteria | Cross-sectional study | 16S rRNA, V4 | observed species | normal weight VS normal weight | 56 | 24.00 (22.00, 27.00) | NA | 31 | 26.00 (24.00, 27.00) | 19.8 |
|  | 2021 | original | China | East | Fecal | Rotterdam criteria | Cross-sectional study | 16S rRNA, V4 | PD whole tree | normal weight VS normal weight | 56 | 24.00 (22.00, 27.00) | NA | 31 | 26.00 (24.00, 27.00) | 19.8 |
|  | 2021 | re-analysis | China | East | Fecal | Rotterdam criteria | Cross-sectional study | 16S rRNA, V4 | Ace index | normal weight VS normal weight | 56 | 24.00 (22.00, 27.00) | 21.07 | 31 | 26.00 (24.00, 27.00) | 19.8 |
|  | 2021 | re-analysis | China | East | Fecal | Rotterdam criteria | Cross-sectional study | 16S rRNA, V4 | Shannon index | normal weight VS normal weight | 56 | 24.00 (22.00, 27.00) | 21.07 | 31 | 26.00 (24.00, 27.00) | 19.8 |
|  | 2021 | re-analysis | China | East | Fecal | Rotterdam criteria | Cross-sectional study | 16S rRNA, V4 | Simpson index | normal weight VS normal weight | 56 | 24.00 (22.00, 27.00) | 21.07 | 31 | 26.00 (24.00, 27.00) | 19.8 |
|  | 2021 | original | China | East | Fecal | Rotterdam criteria | Cross-sectional study | 16S rRNA, V4 | Chao1 | normal weight VS normal weight | 56 | 24.00 (22.00, 27.00) | NA | 31 | 26.00 (24.00, 27.00) | 19.8 |
| Lüll | 2021 | original | Finland | West | Fecal | Rotterdam criteria | Prospective, case–control study | 16S rRNA, V3-4 | Shannon index | PCOS women VS healthy women | 102 | NA | 26.96 | 201 | NA | 25.09 |
| Parodi | 2021 | original | North America | West | Fecal | NIH | Cross-sectional study | 16S rRNA, V? | Observed OTUs | PCOS women VS healthy women | 23 | 29.0 ± 5 | 33.5 | 23 | 35.0 ± 7 | 26.9 |
| Mammadova | 2021 | original | Turkey | West | Fecal | Rotterdam criteria | Cross-sectional study | 16S rRNA, V3-4 | PD whole tree | normal weight VS normal weight | 24 | 19.5 (19.0-22.5) | 22.9 | 22 | 23.0 (22.0-24.3) | 22.5 |
|  | 2021 | original | Turkey | West | Fecal | Rotterdam criteria | Cross-sectional study | 16S rRNA, V3-4 | Shannon index | normal weight VS normal weight | 24 | 19.5 (19.0-22.5) | 22.9 | 22 | 23.0 (22.0-24.3) | 22.5 |
|  | 2021 | original | Turkey | West | Fecal | Rotterdam criteria | Cross-sectional study | 16S rRNA, V3-4 | Observed OTUs | normal weight VS normal weight | 24 | 19.5 (19.0-22.5) | 22.9 | 22 | 23.0 (22.0-24.3) | 22.5 |
| Dong | 2021 | original | China | East | Fecal | Rotterdam criteria | Cross-sectional study | 16S rDNA Full-Length | Shannon index | PCOS women VS healthy women | 45 | 30(27-34) | 26.95 | 37 | 31(28-33.5) | 22.58 |
|  | 2021 | original | China | East | Fecal | Rotterdam criteria | Cross-sectional study | 16S rDNA Full-Length | Shannon index | insulin resistance VS insulin resistance | 27 | 30(26.75-32.25) | 29.29 | 7 | 31(28-32) | 25.4 |
|  | 2021 | original | China | East | Fecal | Rotterdam criteria | Cross-sectional study | 16S rDNA Full-Length | Shannon index | non-insulin resistance VS non-insulin resistance | 15 | 28(26-36) | 22.1 | 22 | 32(29.5-34) | 22.45 |
|  | 2021 | original | China | East | Fecal | Rotterdam criteria | Cross-sectional study | 16S rDNA Full-Length | Shannon index | overweight VS overweight | 31 | 30(27-33) | 29.03 | 12 | 31(28.5-32.25) | 25.63 |
|  | 2021 | original | China | East | Fecal | Rotterdam criteria | Cross-sectional study | 16S rDNA Full-Length | Shannon index | normal weight VS normal weight | 14 | 30.5(25.75-35) | 21.83 | 25 | 31(28-35) | 21.28 |
| Zhu | 2021 | original | China | East | Fecal | Rotterdam criteria | Cross-sectional study | 16S rRNA, V3-4 | Shannon index | PCOS women VS healthy women | 54 | NA | NA | 33 | NA | NA |
| Zhou | 2021 | re-analysis | China | East | Fecal | Rotterdam criteria | Cross-sectional study | 16S rRNA, V1-2 | Chao1 | obese VS obese | 18 | 26±4 | 29.78 | 15 | 24±1 | 29.72 |
|  | 2021 | re-analysis | China | East | Fecal | Rotterdam criteria | Cross-sectional study | 16S rRNA, V1-2 | Observed OTUs | obese VS obese | 18 | 26±4 | 29.78 | 15 | 24±1 | 29.72 |
|  | 2021 | original | China | East | Fecal | Rotterdam criteria | Cross-sectional study | 16S rRNA, V1-2 | Sobs index | obese VS obese | 18 | 26±4 | 29.78 | 15 | 24±1 | 29.72 |
|  | 2021 | original | China | East | Fecal | Rotterdam criteria | Cross-sectional study | 16S rRNA, V1-2 | Shannon index | obese VS obese | 18 | 26±4 | 29.78 | 15 | 24±1 | 29.72 |
|  | 2021 | re-analysis | China | East | Fecal | Rotterdam criteria | Cross-sectional study | 16S rRNA, V1-2 | Ace index | obese VS obese | 18 | 26±4 | 29.78 | 15 | 24±1 | 29.72 |
|  | 2021 | re-analysis | China | East | Fecal | Rotterdam criteria | Cross-sectional study | 16S rRNA, V1-2 | observed species | obese VS obese | 18 | 26±4 | 29.78 | 15 | 24±1 | 29.72 |
|  | 2021 | re-analysis | China | East | Fecal | Rotterdam criteria | Cross-sectional study | 16S rRNA, V1-2 | Simpson index | obese VS obese | 18 | 26±4 | 29.78 | 15 | 24±1 | 29.72 |
|  | 2021 | re-analysis | China | East | Fecal | Rotterdam criteria | Cross-sectional study | 16S rRNA, V1-2 | PD whole tree | obese VS obese | 18 | 26±4 | 29.78 | 15 | 24±1 | 29.72 |
| Hassan | 2022 | original | India | East | Fecal | Rotterdam criteria | Cross-sectional study | 16S rRNA, V3-4 | Shannon index | PCOS women VS healthy women | 19 | 23.9 (6.9) | 25.4 | 20 | 21.1 (2.5) | 23.2 |
| He | 2021 | original | China | East | Fecal | Rotterdam criteria | Cross-sectional study | 16S rRNA, V3-4 | Chao1 | normal weight VS normal weight | 10 | 26.4 ± 3.41 | 21.32 | 12 | 28.25 ± 1.22 | 21.28 |
|  | 2021 | original | China | East | Fecal | Rotterdam criteria | Cross-sectional study | 16S rRNA, V3-4 | Chao1 | normal weight VS normal weight | 14 | 26.71 ± 2.43 | 21.91 | 12 | 28.25 ± 1.22 | 21.28 |
|  | 2021 | original | China | East | Fecal | Rotterdam criteria | Cross-sectional study | 16S rRNA, V3-4 | Observed OTUs | normal weight VS normal weight | 10 | 26.4 ± 3.41 | 21.32 | 12 | 28.25 ± 1.22 | 21.28 |
|  | 2021 | original | China | East | Fecal | Rotterdam criteria | Cross-sectional study | 16S rRNA, V3-4 | Observed OTUs | normal weight VS normal weight | 14 | 26.71 ± 2.43 | 21.91 | 12 | 28.25 ± 1.22 | 21.28 |
| Garcia-Beltran | 2020 | re-analysis | Catalan | West | Fecal | Rotterdam criteria | Cross-sectional study | 16S rRNA, V3-4 | Observed OTUs | PCOS women VS healthy women | 29 | 15.8 | 25 | 31 | 15.9 | 22 |
|  | 2020 | re-analysis | Catalan | West | Fecal | Rotterdam criteria | Cross-sectional study | 16S rRNA, V3-4 | Chao1 | PCOS women VS healthy women | 29 | 15.8 | 25 | 31 | 15.9 | 22 |
|  | 2020 | original | Catalan | West | Fecal | Rotterdam criteria | Cross-sectional study | 16S rRNA, V3-4 | amplicon sequence variants | PCOS women VS healthy women | 29 | 15.8 | 25 | 31 | 15.9 | 22 |
|  | 2020 | original | Catalan | West | Fecal | Rotterdam criteria | Cross-sectional study | 16S rRNA, V3-4 | Pielou's Evenness | PCOS women VS healthy women | 29 | 15.8 | 25 | 31 | 15.9 | 22 |
|  | 2020 | original | Catalan | West | Fecal | Rotterdam criteria | Cross-sectional study | 16S rRNA, V3-4 | Shannon index | PCOS women VS healthy women | 29 | 15.8 | 25 | 31 | 15.9 | 22 |
|  | 2020 | re-analysis | Catalan | West | Fecal | Rotterdam criteria | Cross-sectional study | 16S rRNA, V3-4 | observed species | PCOS women VS healthy women | 29 | 15.8 | 25 | 31 | 15.9 | 22 |
|  | 2020 | re-analysis | Catalan | West | Fecal | Rotterdam criteria | Cross-sectional study | 16S rRNA, V3-4 | Simpson index | PCOS women VS healthy women | 29 | 15.8 | 25 | 31 | 15.9 | 22 |
|  | 2020 | re-analysis | Catalan | West | Fecal | Rotterdam criteria | Cross-sectional study | 16S rRNA, V3-4 | PD whole tree | PCOS women VS healthy women | 29 | 15.8 | 25 | 31 | 15.9 | 22 |
| Liang | 2021 | original | China | East | Fecal | Rotterdam criteria | Cross-sectional study | 16S rRNA, V4 | Chao1 | normal weight VS normal weight | 10 | 24.13 ± 2.45 | 20.46 | 10 | 25.08 ± 3.59 | 20.43 |
|  | 2021 | original | China | East | Fecal | Rotterdam criteria | Cross-sectional study | 16S rRNA, V4 | Chao1 | overweight VS overweight | 10 | 28.94 ± 6.13 | 27.34 | 10 | 30.12 ± 5.20 | 26.05 |
|  | 2021 | re-analysis | China | East | Fecal | Rotterdam criteria | Cross-sectional study | 16S rRNA, V4 | Observed OTUs | overweight VS overweight | 10 | 28.94 ± 6.13 | 27.34 | 10 | 30.12 ± 5.20 | 26.05 |
|  | 2021 | re-analysis | China | East | Fecal | Rotterdam criteria | Cross-sectional study | 16S rRNA, V4 | Observed OTUs | normal weight VS normal weight | 10 | 24.13 ± 2.45 | 20.46 | 10 | 25.08 ± 3.59 | 20.43 |
|  | 2021 | original | China | East | Fecal | Rotterdam criteria | Cross-sectional study | 16S rRNA, V4 | observed species | overweight VS overweight | 10 | 28.94 ± 6.13 | 27.34 | 10 | 30.12 ± 5.20 | 26.05 |
|  | 2021 | original | China | East | Fecal | Rotterdam criteria | Cross-sectional study | 16S rRNA, V4 | observed species | normal weight VS normal weight | 10 | 24.13 ± 2.45 | 20.46 | 10 | 25.08 ± 3.59 | 20.43 |
|  | 2021 | re-analysis | China | East | Fecal | Rotterdam criteria | Cross-sectional study | 16S rRNA, V4 | Simpson index | overweight VS overweight | 10 | 28.94 ± 6.13 | 27.34 | 10 | 30.12 ± 5.20 | 26.05 |
|  | 2021 | re-analysis | China | East | Fecal | Rotterdam criteria | Cross-sectional study | 16S rRNA, V4 | PD whole tree | overweight VS overweight | 10 | 28.94 ± 6.13 | 27.34 | 10 | 30.12 ± 5.20 | 26.05 |
|  | 2021 | original | China | East | Fecal | Rotterdam criteria | Cross-sectional study | 16S rRNA, V4 | Ace index | overweight VS overweight | 10 | 28.94 ± 6.13 | 27.34 | 10 | 30.12 ± 5.20 | 26.05 |
|  | 2021 | original | China | East | Fecal | Rotterdam criteria | Cross-sectional study | 16S rRNA, V4 | Ace index | normal weight VS normal weight | 10 | 24.13 ± 2.45 | 20.46 | 10 | 25.08 ± 3.59 | 20.43 |
|  | 2021 | original | China | East | Fecal | Rotterdam criteria | Cross-sectional study | 16S rRNA, V4 | Shannon index | overweight VS overweight | 10 | 28.94 ± 6.13 | 27.34 | 10 | 30.12 ± 5.20 | 26.05 |
|  | 2021 | original | China | East | Fecal | Rotterdam criteria | Cross-sectional study | 16S rRNA, V4 | Shannon index | normal weight VS normal weight | 10 | 24.13 ± 2.45 | 20.46 | 10 | 25.08 ± 3.59 | 20.43 |
|  | 2021 | re-analysis | China | East | Fecal | Rotterdam criteria | Cross-sectional study | 16S rRNA, V4 | Simpson index | normal weight VS normal weight | 10 | 24.13 ± 2.45 | 20.46 | 10 | 25.08 ± 3.59 | 20.43 |
|  | 2021 | re-analysis | China | East | Fecal | Rotterdam criteria | Cross-sectional study | 16S rRNA, V4 | PD whole tree | normal weight VS normal weight | 10 | 24.13 ± 2.45 | 20.46 | 10 | 25.08 ± 3.59 | 20.43 |
| Haudum | 2020 | original | Austria | West | Fecal | Rotterdam criteria | Pilot cohort | 16S rRNA, V1-2 | PD whole tree | PCOS women VS healthy women | 24 | median = 27, IQR = 5.9 | 24.9 | 20 | median = 32, IQR = 12.0 | 22.3 |
| Wang | 2023 | original | China | East | Fecal | Rotterdam criteria | Cross-sectional study | 16S rRNA, V4 | observed species | PCOS women VS healthy women | 24 | 29.92 ± 3.39 | n/a | 24 | 31.00 ± 2.96 | n/a |
|  | 2023 | original | China | East | Fecal | Rotterdam criteria | Cross-sectional study | 16S rRNA, V4 | Shannon index | PCOS women VS healthy women | 24 | 29.92 ± 3.39 | n/a | 24 | 31.00 ± 2.96 | n/a |
|  | 2023 | original | China | East | Fecal | Rotterdam criteria | Cross-sectional study | 16S rRNA, V4 | Chao1 | PCOS women VS healthy women | 24 | 29.92 ± 3.39 | n/a | 24 | 31.00 ± 2.96 | n/a |
|  | 2023 | original | China | East | Fecal | Rotterdam criteria | Cross-sectional study | 16S rRNA, V4 | Ace index | PCOS women VS healthy women | 24 | 29.92 ± 3.39 | n/a | 24 | 31.00 ± 2.96 | n/a |
| Yin | 2022 | original | china | East | Fecal | Rotterdam criteria | Cross-sectional study | 16S rRNA, V3-4 | Chao1 | overweight VS overweight | 25 | 29.04 ± 3.89 | 28.19 | 20 | 29.9 ± 2.98 | 29.03 |
|  | 2022 | original | china | East | Fecal | Rotterdam criteria | Cross-sectional study | 16S rRNA, V3-4 | Shannon index | overweight VS overweight | 25 | 29.04 ± 3.89 | 28.19 | 20 | 29.9 ± 2.98 | 29.03 |
|  | 2022 | original | china | East | Fecal | Rotterdam criteria | Cross-sectional study | 16S rRNA, V3-4 | Chao1 | normal weight VS normal weight | 25 | 28.96 ± 3.87 | 20.68 | 20 | 28.53 ± 3.19 | 20.01 |
|  | 2022 | original | china | East | Fecal | Rotterdam criteria | Cross-sectional study | 16S rRNA, V3-4 | Shannon index | normal weight VS normal weight | 25 | 28.96 ± 3.87 | 20.68 | 20 | 28.53 ± 3.19 | 20.01 |
| Yu | 2022 | original | china | East | Fecal | Rotterdam criteria | Cross-sectional study | 16S rRNA, V3-4 | Chao1 | PCOS women VS healthy women | 20 | 28.95 ± 5.83 | 23.81 | 20 | 26.75 ± 5.46 | 22.54 |
|  | 2022 | original | china | East | Fecal | Rotterdam criteria | Cross-sectional study | 16S rRNA, V3-4 | Shannon index | PCOS women VS healthy women | 20 | 28.95 ± 5.83 | 23.81 | 20 | 26.75 ± 5.46 | 22.54 |
| Yang | 2022 | original | china | East | Fecal | Rotterdam criteria | Cross-sectional study | METAGENOMICS | Shannon index | PCOS women VS healthy women | 32 | 29.34 ± 2.88 | 23.46 | 18 | 29.89 ± 3.36 | 20.44 |
|  | 2022 | original | china | East | Fecal | Rotterdam criteria | Cross-sectional study | METAGENOMICS | Simpson index | PCOS women VS healthy women | 32 | 29.34 ± 2.88 | 23.46 | 18 | 29.89 ± 3.36 | 20.44 |

NA, no information

Supplemental Table 3: Stool sample processing methods in the included studies.

| **Study** | **Collection & handling by participant** | **Long-term storage** | **DNA extraction method** |
| --- | --- | --- | --- |
| Liang, et al. 2020 | not mentioned | Samples were immediatelly frozen and stored at -80°C until analysis. | DNA was extracted from stool samples with E.Z.N.A. stool DNA kit (Omega Biotek, Norcross, GA, USA). |
| Zhou, et al. 2020 | Each participant was collected during non- menstrual period and transferred to the laboratory in an ice box within 2 h. | Each stool sample was separately loaded into 4 EP tubes and frozen at −80°C until use. | Total DNA of the gut microbiota was obtained using the EZNA® soil kit (Omega Bio-tek, Norcross, GA, USA). |
| Jobioa, et al. 2020 | Stool samples were collected at home the day prior to blood sampling using stool collection tubes and frozen in the participant freezer. | Upon return to study staff, samples were stored at -80℃ until further processing. | DNA was extracted from 50-100mg stool samlpe using the PowerFecal DNA isolation kit (QIAamp Powerfecal DNA kit (Qiagen INC, Hilden, Germany). |
| zeng, et al. 2019 | not mentioned | not mentioned | Total DNA was extracted from each stool sample using a QIAamp DNA stool mini kit (Qiagen 51,504, Germany) |
| Qi, et al. 2019 | not mentioned | not mentioned | A frozen aliquot (200mg) of each fecal sample was processed using the Stool Genomic DNA Kit (CW2092S; CWBIO). |
| Torres, et al. 2018 | not mentioned | Samples were stored at -80°C within 20 minutes of collection. | Genomic DNA was extracted from samples in a class II biological safety cabinet using the PowerSoil DNA Isolation Kit (MoBio Lab- oratories, Inc.). |
| Insenser, et al. 2018 | Patients were instructed to collect fecal samples into sterile plastic bottle topped with a screw cap. | The samples were immediately divided into aliquots and stored at -80°C after collection until analysis was performed. Samples were defrosted slowly at 4°C to avoid DNA degradation and further thawed at room temperature. | Total DNA from 1 mL of the supernatant was obtained with the QIAamp DNA Mini KitPellet commercial kit (Qiagen, Hilden, Germany). |
| Liu, et al. 2017 | not mentioned | Feces was divided into aliquots and was frozen on dry ice immediately upon collection and stored at −80◦ C until analysis. | DNA extraction from each frozen fecal sample was conducted with guanidine thiocyanate–0.1 M Tris (pH 7.5) and 600 ul of 10% N-lauroyl sar- cosine |
| Lindheim, et al. 2017 | Stool samples were collected using empty stool collection tubes with an inbuilt spatula (Praxisdienst GmbH, Longuich, Germany). | Samples were stored short-term at -20 ̊C (mean ±SD = 4±1.1 days) and then at -70 ̊C until further processing. | Total DNA was extracted from stool samples using the MagNA Pure LC DNA Isolation Kit III (Bacteria, Fungi) (Roche, Rotkreuz, Switzerland) according to the manufacturer’s instructions with additional bead-beating with MagNA Lyser Green Beads (Roche) and lysozyme treatment (Roth, Karlsruhe, Germany). |
| Eyupoglu, et al. 2020 | Approximately five grams of fecal samples were collected into plastic screw-cap containers | Stored at -80℃ within half an hour. | DNA extraction was performed using Qiagen Stool Mini Kit (Qiagen, Hilden, Germany), as directed by the manufacturer. |
| Yang et al. 2021 | Stool samples were collected using stool collection tubes. | Stored in a -80°C freezer. | A frozen aliquot (200 mg) of each fecal sample was processed using the PowerSoil DNA Extraction Kit (Shenzhen Bioeasy Biotechnologies Co., Ltd., China). |
| Lüll et al. 2021 | Fecal samples were collected at home by the study participants. It was recommended that the fecal sample should be delivered in a cooler on the day of collection. If that was not possible, the sample was stored for 1 or 2 days in a freezer at –20°C until delivery. | After delivery, the fecal samples were initially stored at –20°C and then moved to –70°C for long-term storage. | The samples were homogenized in a Stomacher-400 blender. The QIAamp Stool Mini Kit (Qiagen, Venlo, The Netherlands) was used for DNA extraction. |
| Parodi et al. 2021 | not mentioned | not mentioned | MicrobiaDNA was isolated from stool with Qiagen Magattract PowermicrobiomeDNA/RNA KF kits |
| Mammadova et al. 2021 | Stool samples were collected at the Molecular Microbiology Laboratory of the Hacettepe University. Two hundred mg of each stool sample was placed into Eppendorf tubes. | All stool samples taken from the PCOS and control groups were stored at −80°C until the day of study. | DNA was extracted from faecal material using a stool extrac- tion kit according to the manufacturers’ instructions (Qiagen, CA). |
| Dong et al. 2021 | Fecal samples were collected in the morning during each non- menstrual period. | Patients were instructed to collect fecal samples into fecal DNA storage tubes (CW2654, CwBiotech, Beijing, China) and then the samples were stored and sent to laboratory under room temperature. | Bacterial DNA was extracted using an intestinal DNA extraction kit (Qiagen Fecal DNA Extraction Kit, Qiagen, Hilden, Germany). |
| Zhu et al. 2021 | Blood samples were collected on any day of the menstrual cycle. | About 3–5 g of fresh feces was collected from each participant during the non-menstrual period and transferred to the laboratory’s refrigerator within 2 h (keeping the temperature below 4°C), and then frozen at −80°C until detection. | The total DNA was extracted from each fecal sample using the QIAamp DNA fecal micro-Kit (Qiagen 51504, Germany) and treated with RNase A at 37°C for 7 min (final concentration of 0.1 mg/ml) before digestion by proteinase K digestion. |
| Zhou et al. 2020 | A sterile plastic spoon and tube were used to collect approximately 10g of fresh fecal sample from each subject. | The samples were placed in an ice box, transported to the laboratory within 2h, and then stored at −80◦C. | Total DNA was extracted from stool samples using the MagNA Pure LC DNA Isolation Kit III (Bacteria, Fungi) (Roche, Rotkreuz, Switzerland) with additional bead-beating with MagNA Lyser Green Beads (Roche) and lysozyme treatment (Roth, Karlsruhe, Germany). |
| Hassan et al. 2022 | Each participant was asked to provide fresh stool sample (approx. 5 g) at the same day with blood collection, by using the stool collection and stabilization kit (OMNIgene®•GUT OMR-200, DNA Genotek, Canada). | Stored at −80◦C. | DNA extraction was performed by as per the instruction manual using ZymoBIOMICSTM DNA kit by Zymo Research USA. |
| He et al. 2021 | not mentioned | Feces samples were collected from all patients after menstruation, and the samples were immediately frozen and stored at -80°C until analysis. | Microbial DNA was extracted from stool samples using a TIANgen stool DNA kit. |
| Zhang, et al. 2019 | Fecal samples were collected from each individual in the morning before their first meal. | After the weight of the fecal sample was determined, a sample protector (CWBIO, China) was added at a ratio of one-part fecal sample to five parts of the sample protector, after which the samples were stored at -20°C until further processing. | A QIAamp DNA stool minikit (Qiagen, Hilden, Germany) was used for metagenomic DNA extraction. |
| Garcia-Beltran et al. 2020 | Stool samples were collected at home using a faeces collector (FECOTAINER, Excretas Medical BV, Enschede, The Netherlands) in the morning of the day before the hospital appointment. | Samples were frozen in the patient's freezer at −20°C and transported on ice to the hospital, where they were sprayed with liquid nitrogen, aliquoted and stored at −80°C until analysis. | DNA was extracted using MagMAX CORE Nucleic method. Acid Purification Kit 500 RXN (Thermo Fisher, CA, Austin), following the manufacturer's instructions. |
| Liang et al. 2021 | Fecal samples were collected into sterile plastic bottle in non-menstrual period and immediately divided into aliquots within 20 min. | Stored at − 80 °C until analysis was per- formed. | DNA extraction was conducted using a E.Z.N.A.® Stool DNA Kit (Omega Bio-tek, GA, USA) according to the manufacturer’s instructions. |
| Chu et al. 2020 | Fresh fecal samples were collected from the participants on their first visit and before any pharmacotherapy treatment. at the Centre for Reproductive Medicine. | Fecal samples were then transported to the laboratory with an ice pack within 2 hours. All samples were then snap frozen and stored at -80°C before analyses. | DNA from each fecal sample using an improved protocol based on the QIAamp Fast DNA Stool Mini Kit instructions (Qiagen). |
| Haudum et al. 2020 | Stool samples were self-collected before the first soy drink using empty stool collection tubes with an inbuilt spatula (Praxisdienst GmbH, the first soy drink using empty stool collection tubes with an inbuilt spatula (Praxisdienst GmbH, Longuich, Germany). | Fecal samples stored short-term at −16 °C, and returned to the outpatient clinic on cool packs on Longuich, Germany), stored short-term at −16 ◦C, and returned to the outpatient clinic on cool packs the morning following the last soy drink. | Total DNA was extracted from stool samples using the MagNA Pure LC DNA Isolation Kit III (Bacteria, Fungi) on the MagNA Pure Instrument (Roche, Rotkreuz, Switzerland). |
| Wang et al. 2023 | the human subjects voided their feces into a clean collection box after the bladder was emptied, and approximately 20 g of fresh feces from participants | Once the fecal samples were collected, they were immediately transported to Novogene company on dry ice for high-throughput 16 S rDNA sequencing. | All samples were sequenced following the Earth Microbiome Project 16 S Illumina Amplicon Protocol |
| Yin et al. 2022 | All the participants were asked to come to our department during days 2–4 of the menstrual period after an overnight fast. | Not mentioned | DNA from stool samples was extracted using HiPure Stool DNA Kits B (D3141-03B, Guangzhou Meiji Bio- technology Co., Ltd., China). |
| Yu et al. 2022 | Stool samples were obtained from the participants 3–5 days after menstruation. | The blood and fecal sam- ples were transported within 2 h and preserved at − 80 °C until further processing. | Stool samples were thawed in 4 °C water, centrifuged for 10 min, and DNA was extracted using Axygen Axy Prep DNA Gel Kit (Axygen, USA). |
| Yang et al. 2022 | Serum and fecal samples were collected once between the third and fifth day of the menstrual cycle in the maternity center at ZhuJiang Hospital of Southern Medical University | fecal samples were immediately stored at −80°C for further analysis. | A total of 250 mg fecal sample was used for DNA extraction using the PureLinkTM Stool Genomic DNA Kit (ThermoFisher Scientific Inc., Massachusetts, United States), following the manufacturer’s protocol. |

Supplemental Table 4 Publish bias assessment by egger regression test in alpha diversity indexes.

| **Alpha indexes** | **t** | **df** | **p-value** |
| --- | --- | --- | --- |
| Chao 1 | -2.80 | 18 | 0.0118 |
| Observed species | -2.05 | 8 | 0.0740 |
| Shannon index | -0.45 | 26 | 0.6568 |
| Simpson index | -0.28 | 9 | 0.7839 |
| PD whole tree | -1.99 | 8 | 0.0822 |

Supplemental Table 5: Subgroup and meta-regression analysis of α-diversity (Chao 1) difference between PCOS patients and healthy control.

| **Sub-**  **group** | **No. of estimate** | **SMD (95%CI)** | **I^2^** | **Model** | **p-**  **value^*^** | **Meta-regression^#^**  **OR, (95%CI), p-value** | **R^2$^** |
| --- | --- | --- | --- | --- | --- | --- | --- |
| Region | | | | | | |  |
| East | 15 | -0.10 (-0.39, 0.19) | 66% | Random | <0.01 | 1 | 0.00% |
| West | 5 | 0.01 (-0.52, 0.54) | 68% | Random | <0.01 | 0.91, (0.62,2.01), 0.71 |  |
| BMI difference | | | | | | |  |
| No | 17 | -0.10 (-0.39, 0.20) | 69% | Random | <0.01 | 1 | 0.00% |
| Yes | 2 | 0.10 (-0.30, 0.48) | 41% | Fix | 0.19 | 1.16, (0.51, 2.63), 0.73 |  |
| Age difference | | | | | | |  |
| No | 18 | -0.06 (-0.32, 0.20) | 60% | Random | <0.01 | 1 | 0.00% |
| Yes | 2 | -0.09 (-1.46, 1.29) | 91% | Random | <0.01 | 1.16, (0.51, 2.63), 0.73 |  |
| HOMA-IR difference | | | | | | |  |
| No | 10 | 0.02 (-0.27, 0.28) | 39% | Fix | 0.10 | 1 | 0.00% |
| Yes | 4 | -0.18 (-1.20, 0.85) | 92% | Random | <0.01 | 0.92, (0.42, 2.02), 0.83 |  |
| Testosterone difference | | | | | | |  |
| No | 3 | -0.44 (-0.88, 0.00) | 22% | Fix | 0.28 | 1 | 3.36% |
| Yes | 15 | 0.02 (-0.31, 0.28) | 69% | Random | <0.01 | 1.43, (0.68, 2.99), 0.35 |  |
| FSH difference | | | | | | |  |
| No | 12 | -0.15 (-0.53, 0.24) | 75% | Random | <0.01 | 1 | 0.00% |
| Yes | 4 | -0.20 (-0.51, 0.11) | 0 | Fix | 0.64 | 0.94, (0.49, 1.82), 0.76 |  |
| LH difference | | | | | | |  |
| No | 4 | -0.35 (-0.63, 0.15) | 25% | Fix | 0.26 | 1 | 0.00% |
| Yes | 12 | -0.11 (-0.45, 0.24) | 74% | Random | <0.01 | 1.23, (0.62, 2.44), 0.56 |  |
| LH/FSH difference | | | | | | |  |
| No | 3 | -0.73 (-1.78, -0.29) | 0% | Fix | 0.53 | 1 | 22.75% |
| Yes | 12 | -0.01 (-0.34, 0.31) | 69% | Random | <0.01 | 2.19, (1.04, 4.59), 0.04 |  |

* p value for heterogeneity test by DerSimonian–Laird estimator.

# Univariate Meta-regression analysis were performed to explore heterogeneity source.

$ amount of heterogeneity accounted for.

NA, not assessed.

Supplemental Table 6: Subgroup and meta-regression analysis of α-diversity (Observed species) difference between PCOS patients and healthy control.

| **Sub-group** | **No. of estimate** | **SMD (95%CI)** | **I2** | **Model** | **p-value*** | **Meta-regression#**  **OR, 95%CI, p-value** | **R2$** |
| --- | --- | --- | --- | --- | --- | --- | --- |
| Region | | | | | | | |
| East | 8 | -0.10 (-0.33, 0.53) | 65% | Random | <0.01 | 1 | 0.00% |
| West | 2 | -0.21 (-1.24, 0.28) | 58% | Random | <0.01 | 0.75, (0.29, 1.89), 0.54 |  |
| BMI difference | | | | | | | |
| No | 8 | -0.14 (-0.55, 0.27) | 66% | Random | 0.01 | 1 | 0.00% |
| Yes | 1 | 0.30 (-020, 0.81) | NA | NA | NA | 1.56, (0.52, 4.67), 0.42 |  |
| Age difference | | | | | | | |
| No | 9 | -0.14 (-0.23, 0.51) | 60% | Random | 0.01 | 1 | 23.97% |
| Yes | 1 | -0.75 (-1.36, -0.13) | NA | NA | NA | 0.41, (0.14, 1.24), 0.12 |  |
| HOMA-IR difference | | | | | | | |
| No | 5 | 0.33 (-0.03, 0.64) | 38% | Fix | 0.17 | 1 | 0.00% |
| Yes | 4 | -0.15 (-0.89, 0.59) | 84% | Random | <0.01 | 0.68, (0.29, 1.58), 0.37 |  |
| Testosterone difference | | | | | | | |
| No | 3 | -0.46 (-0.89, -0.02) | 0% | Fix | 0.41 | 1 | 3.36% |
| Yes | 7 | 0.20 (-0.24, 0.64) | 67% | Random | <0.01 | 1.83, (0.84, 3.98), 0.12 |  |
| FSH difference | | | | | | | |
| No | 8 | -0.14 (-0.55, 0.27) | 66% | Random | <0.01 | 1 | 28.45% |
| Yes | 1 | 0.93 (0.33, 1.53) | NA | NA | NA | 0.94, (0.49, 1.82), 0.06 |  |
| LH difference | | | | | | | |
| No | 3 | 0.32 (-0.42, 1.07) | 64% | Random | 0.06 | 1 | 0.00% |
| Yes | 6 | -0.17 (-0.68, 0.34) | 75% | Random | <0.01 | 0.61, (0.25, 1.52), 0.28 |  |
| LH/FSH difference | | | | | | | |
| No | 2 | -0.13 (-0.63, 0.37) | 74% | Random | <0.01 | 1 | 0.00% |
| Yes | 6 | -0.22 (-0.85, 0.42) | 0% | Fix | 0.32 | 1.08, (0.38, 3.07), 0.88 |  |

* p value for heterogeneity test by DerSimonian–Laird estimator.

# Univariate Meta-regression analysis were performed to explore heterogeneity source.

$ amount of heterogeneity accounted for.

NA, not assessed.

Supplemental Table 7: Subgroup and meta-regression analysis of α-diversity (PD) difference between PCOS patients and healthy control.

| **Sub-group** | **No. of estimate** | **SMD (95%CI)** | **I^2^** | **Model** | **p-value*** | **Meta-regression#**  **OR, 95%CI, p-value** | **R^2$^** |
| --- | --- | --- | --- | --- | --- | --- | --- |
| Region | | | | | | | |
| East | 6 | -0.28 (-0.79, 0.23) | 68% | Random | <0.01 | 1 | 0.00% |
| West | 4 | -0.53 (-1.01, -0.04) | 63% | Random | 0.04 | 0.77, (0.38, 1.60), 0.49 |  |
| BMI difference | | | | | | | |
| No | 9 | -0.41 (-0.81, -0.01) | 71% | Random | <0.01 | 1 | 0.00% |
| Yes | 1 | -0.28 (-0.79, 0.23) | NA | NA | NA | 1.14, (0.35, 3.64), 0.82 |  |
| Age difference | | | | | | | |
| No | 7 | -0.27 (-0.69, 0.15) | 62% | Random | <0.01 | 1 | 1.54% |
| Yes | 3 | -0.62 (-1.29, 0.04) | 72% | Random | 0.03 | 0.70, (0.33, 1.50), 0.36 |  |
| HOMA-IR difference | | | | | | | |
| No | 5 | -0.20 (-0.51, 0.11) | 88% | Random | <0.01 | 1 | 0.00% |
| Yes | 4 | -0.59 (-1.40, 0.22) | 0% | Fixed | 0.77 | 0.72, (0.32, 1.63), 0.44 |  |
| Testosterone difference | | | | | | | |
| No | 6 | -0.35 (-0.79, 0.09) | 72% | Random | 0.13 | 1 | 0.00% |
| Yes | 3 | -0.51 (-1.17, 0.14) | 51% | Random | <0.01 | 1.17, (0.51, 2.66), 0.70 |  |
| FSH difference | | | | | | | |
| No | 8 | -0.48 (-0.92, -0.03) | 73% | Random | <0.01 | NA | NA |
| Yes | 0 | NA | NA | NA | NA | NA |  |
| LH difference | | | | | | | |
| No | 1 | -0.64 (-1.57, 028) | 77% | Random | <0.01 | 1 | 0.00% |
| Yes | 7 | -0.46 (-0.95, 0.03) | NA | NA | NA | 1.20, (0.27, 5.38), 0.81 |  |
| LH/FSH difference | | | | | | | |
| No | 2 | -0.88 (-1.54, -0.22) | 0% | Fix | 0.47 | 1 | 0.00% |
| Yes | 6 | -0.37 (-089, 0.15) | 78% | Random | <0.01 | 1.67, (0.56, 4.98), 0.36 |  |

* p value for heterogeneity test by DerSimonian–Laird estimator.

# Univariate Meta-regression analysis were performed to explore heterogeneity source.

$ amount of heterogeneity accounted for.

NA, not assessed.

Supplemental Table 8: Sensitivity analyses of α-diversity indices difference between PCOS patients and healthy control.

| **Group** | **No. of estimates** | **SMD (95%CI)** | **Model** | **I^2^ (%)** | **P value** |
| --- | --- | --- | --- | --- | --- |
| **Observed species** | | | | | |
| Removing manually calculated studies | 5 | 0.32 (-0.21, 0.85) | Random | 63% | 0.05 |
| Removing high risk studies | 8 | -0.06 (-0.47, 0.35) | Random | 64% | <0.01 |
| **Chao 1** | | | | | |
| Removing manually calculated studies | 17 | -0.03 (-0.30, 0.25) | Random | 64% | <0.01 |
| Removing high risk studies | 13 | -0.18 (-0.37, 0.01) | Fixed | 22% | 0.22 |
| **Shannon Index** | | | | | |
| Removing manually calculated studies | 24 | -0.26 (-0.37, -0.15) | Fixed | 40% | 0.03 |
| Removing high risk studies | 21 | -0.30 (-0.41, -0.18) | Fixed | 39% | 0.04 |
| **Simpson Index** | | | | | |
| Removing manually calculated studies | 3 | -0.02 (-0.75, 0.71) | Random | 76% | 0.01 |
| Removing high risk studies | 10 | -0.19 (-0.40, 0.02) | Fixed | 38% | 0.10 |
| **PD whole tree** | | | | | |
| Removing manually calculated studies | 4 | -0.32 (-0.49, 0.42) | Random | 86% | <0.01 |
| Removing high risk studies | 9 | -0.50 (-0.73, -0.27) | Fixed | 30% | 0.18 |

Supplemental Table 9: Summary of various measures of beta-diversity.

Significant difference; No difference; * Not mentioned.

Supplemental Table 10: Univariate logistic-regression analysis of factors potentially associated with Beta-diversity.

| **Characteristics** | **Group** | **No. of study** | **OR (95%CI), P value** |
| --- | --- | --- | --- |
| Region | East | 24 | 1 |
|  | West | 11 | 0.97 (0.19-4.94), 0.98 |
| BMI difference | Yes | 5 | 1 |
|  | No | 27 | 1.90 (0.26, 13.87), 0.53 |
| Age difference | Yes | 5 | 1 |
|  | No | 28 | 1.67 (0.23-11.92), 0.61 |
| HOMA-IR difference | Yes | 9 | 1 |
|  | No | 8 | 2.05 (0.67-16.51), 0.46 |
| Testosterone difference | Yes | 14 | 1 |
|  | No | 5 | 2.25 (0.21, 23.32), 0.50 |
| FSH difference | Yes | 6 | 1 |
|  | No | 21 | 6.4 (0.89-45.99), 0.06 |
| LH difference | Yes | 23 | 1 |
|  | No | 4 | 1.60 (0.14-18.00), 0.70 |
| LH/FSH difference | Yes | 6 | 1 |
|  | No | 21 | 2.31 (0.22-24.29), 0.70 |

Supplemental Table 10. Summaries of the bacterial function description from the included studies.

| **Qi, et al. 2019** | **Zhang, et al. 2019** | **Chu, et al. 2020** | **Yang, et al. 2022** |
| --- | --- | --- | --- |
| ***Folate biosynthesis;***  ***Glycerophospholipid metabolism;***  ***Biotin metabolism;***  Methane metabolism;  D-arginine and D-ornithine metabolism;  Purine metabolism;  Alanine, aspartate and glutamate;  Sulfur metabolism;  Secondary bile acid biosynthesis;  Steroid hormone biosynthesis; | ***Cationic antimicrobial peptide (CAMP) resistance;***  ***Lipopolysaccharide biosynthesis;***  ***The phosphotransferase system (PTS);***  ***Biotin metabolism;***  ***Folate biosynthesis;***  ***One carbon pool by folate;***  ***Fatty acid biosynthesis;***  Fructose and mannose metabolism;  the citrate cycles;  Flagellar assembly, bacterial chemotaxis;  Thiamine metabolism;  Valine, leucine and isoleucine biosynthesis;  Propanoate metabolism;  ABC transporters;  Bacterial secretion systems; | ***Cationic antimicrobial peptide resistance;***  ***Phosphotransferase system (PTS);***  ***Glycerophospholipid metabolism;***  ***Lipopolysaccharide biosynthesis;***  Pyruvate metabolism;  Glutathione metabolism;  Short-chain fatty acids metabolism;  Glycolysis/gluconeogenesis; | ***Biotin metabolism; Fatty acid metabolism;***  ***One carbon pool by folate;***  Insulin resistance; Insulin signaling pathway;  Endocytosis; Non-alcoholic fatty liver disease;  Selenocompound metabolism;  HIF-1 signaling pathway; Mitophagy;  mTOR signaling pathway;  Phospholipase D signaling pathway;  Breast cancer; EGFR tyrosine kinase inhibitor resistance;  Glioma; ErbB signaling pathway;  Acute myeloid leukemia; Choline metabolism in cancer;  JAK-STAT signaling pathway;  Thyroid hormone signaling pathway;  Endocrine resistance; Longervity regulating pathway;  Longervity regulating pathway-multiple species;  Pathways in cancer; Prostate cancer;  PI3K-Akt signaling pathway; MAPK signaling pathway;  Chloroalkane and chloroallkene degradation;  Carbon fixation pathways in prokaryotes;  Porphyrin and chlorophyⅡ metabolism;  Phenylalanine, tyrosine and tryptophan biosynthesis; Riboflavin metabolism; Cell cycle-Caulobacter;  beta-Lactam resistance |


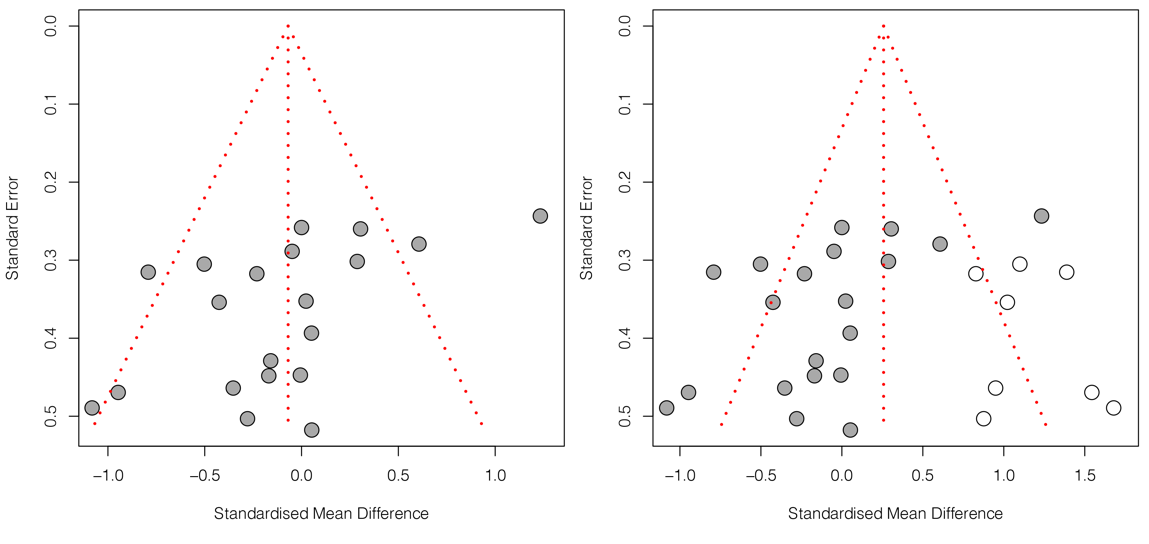


Supplemental Figure 1. Funnel plot (A) and trim-and-fill funnel plot (B) assessing publication bias in the meta-analyses of Chao 1 index.

Supplemental Figure 2. Forest plot by trim-and-fill analysis of Chao 1.


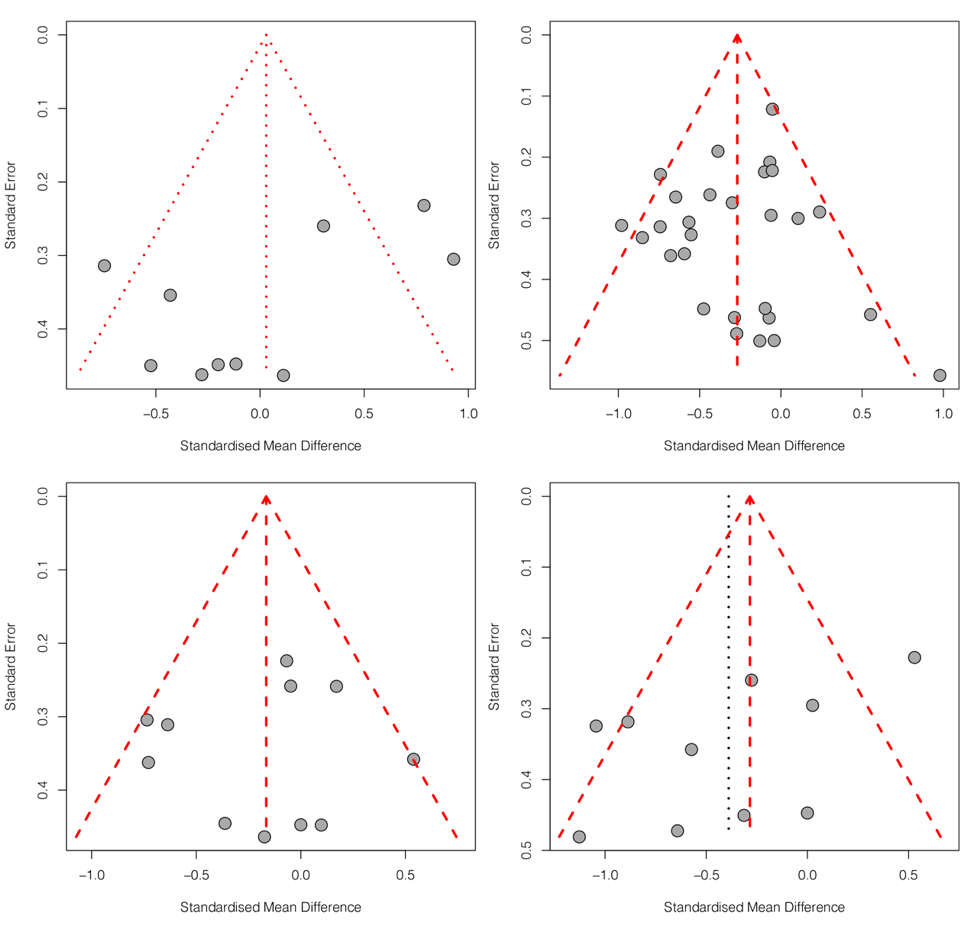


Supplemental Figure 3. Funnel plot of assessing publication bias in the meta-analyses of observed species (A), Shannon (B), Simpson (C), and PD whole tree indexes (D).
